# Antibiotic class-dependent degradation mechanisms and resistance gene elimination by dielectric barrier discharge cold plasma technology for wastewater treatment

**DOI:** 10.1039/d6ra04146a

**Published:** 2026-09-18

**Authors:** Muhammad Ishtiaq Ali, Rameesha Abid, Arzoo Shahzad, Javeria Shakeel, Muhammad Shafiq, Asif Jamal, Hassan Waseem

**Affiliations:** a Department of Microbiology, Quaid-i-Azam University Islamabad 44100 Pakistan ishimrl@qau.edu.pk rameeshaabid@bs.qau.edu.pk arzooshahzad26@gmail.com jvrshkl@gmail.com asifjamal@qau.edu.pk; b Department of Physics, Quaid-i-Azam University Islamabad 44100 Pakistan; c Department of Civil & Environmental Engineering, Carleton University 1125 Colonel By Drive Ottawa ON K1S5B6 Canada hassanwaseem@cunet.carleton.ca ishimrl@qau.edu.pk

## Abstract

Antibiotic residues persisting in aquatic systems at sub-inhibitory concentrations actively drive the spread of antibiotic resistance genes (ARGs), contributing to 1.27 million deaths annually. Conventional treatment methods achieve only 6–37% antibiotic removal and fail to eliminate resistance determinants, establishing the need for advanced alternatives. Dielectric barrier discharge (DBD) cold plasma generates reactive oxygen and nitrogen species (RONS) at atmospheric pressure, enabling the degradation of parent antibiotics and the reduction of ARG abundance in a single treatment. Each antibiotic class degrades through a structurally distinct mechanism: β-lactams undergo rapid ring opening, fluoroquinolones require synergistic oxidative-reductive defluorination, tetracyclines follow stepwise hydroxylation and deamination, and sulfonamides and macrolides primarily undergo S–N and lactone bond cleavage. Removal efficiencies exceeding 90% have been reported for fluoroquinolones, tetracyclines and sulfonamides within 10–60 min under optimized conditions in deionized water; however, these efficiencies may be substantially lower in complex real wastewater matrices. For ARG elimination, DBD plasma achieves up to 15-log reductions in the qPCR-measured abundance of blaTEM-1, aac(3)-II, tetC, tetW, and intI-1 through direct DNA strand breakage and antibiotic-resistant bacteria (ARB) inactivation, though intracellular ARGs (i-ARGs) require 2.5-fold higher plasma energy doses than their extracellular counterparts. This review introduces a class-dependent plasma antibiotic reactivity mapping framework, critically evaluates reactor configurations and process optimization, and highlights the urgent need for studies at environmentally realistic concentrations to bridge the gap between laboratory performance and real-world application.

## Abstract 2

Dielectric barrier discharge cold plasma degrades antibiotics *via* class-specific mechanisms and eliminates resistance genes through RONS-mediated DNA damage, achieving >90% antibiotic removal and up to 15-log ARG reduction in wastewater.

## Graphical abstract

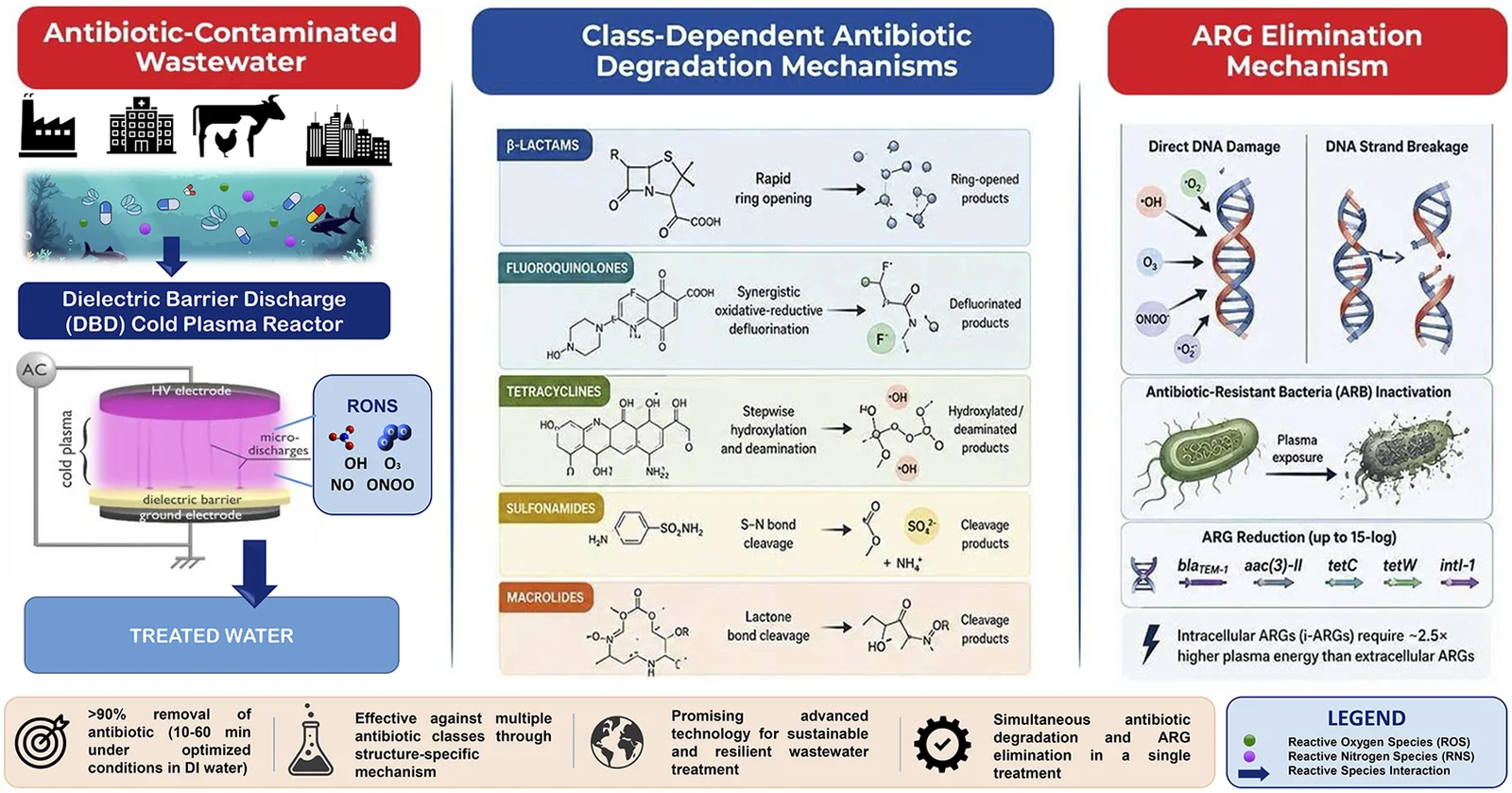

## 1. Introduction

Antibiotic residues have subtly emerged as a major global environmental hazard. These compounds, once vital to modern medicine, now persist in rivers, groundwater and wastewater systems around the world at concentrations high enough to threaten One Health initiatives.^1,2^ Between 2015 and 2025, global antibiotic consumption increased by approximately 46%, driven by expanded use in human healthcare, livestock production and aquaculture.^3^ Contamination enters aquatic systems from four primary source categories: pharmaceutical manufacturing plants, which discharge untreated or poorly treated effluents at concentrations in the mg L^−1^ range; hospital effluents, which carry significant quantities of unmetabolized pharmaceuticals, including ciprofloxacin and sulfamethoxazole at up to hundreds of µg L; agricultural and livestock wastewater and domestic sewage systems. A recent meta-analysis reported median antibiotic concentrations of roughly 20–30 ng L^−1^ in rivers,^4^ while hotspots downstream of hospital or pharmaceutical discharges have registered levels in the tens to hundreds of µg L^−1^.^5^ The highest environmental burdens are concentrated in geographic hotspots in South Asia, China and sub-Saharan Africa, where high antibiotic consumption and limited wastewater treatment converge. Critically, these residues at sub-inhibitory concentrations remain biologically active and promote the proliferation of antibiotic resistance genes (ARGs), accelerating antimicrobial resistance, which causes 1.27 million deaths annually and is projected to cause 10 million deaths annually by 2050.^6,7^

Most conventional wastewater treatment plants were designed for bulk organic waste removal rather than trace pharmaceutical contaminants, and thus, antibiotics pass through largely untreated, with reported removal efficiencies of only 6–37% depending on treatment type and operational conditions.^8^ The common antibiotic classes, such as fluoroquinolones, macrolides and sulfonamides, have removal rates of approximately 40%, thus leaving large residues in effluents.^9^ Several physical treatment methods, including filtration, coagulation–flocculation, adsorption, precipitation, sedimentation, and ion exchange, have conventionally been used for treating wastewater,^10^ but the majority of them are inefficient in the removal of trace-level contaminants, including antibiotics.^11^ Biological systems, such as membrane bioreactors (MBRs), moving-bed-biofilm reactors (MBBRs), anaerobic digestion (AD), integrated fixed-film-activated sludge (IFAS) and aerobic granular sludge (AGS), have been designed to treat antibiotics but result in incomplete degradation and the active enrichment of ARGs within the sludge matrix.^12^ Chemical oxidation methods, including chlorination, ozonation, and photocatalysis,^13,14^ partially degrade antibiotic structures but produce toxic by-products, require high energy inputs and depend on ultraviolet (UV) penetration, making them unsuitable for complex real wastewater matrices.

Advanced oxidation processes (AOPs) have emerged as a third-generation solution, generating highly reactive hydroxyl radicals (˙OH) *in situ* capable of non-selective oxidative degradation of recalcitrant organics. Established AOPs, including UV/H_2_O_2_ (hydrogen peroxide), Fenton oxidation and ozonation, improve removal efficiencies but remain constrained by chemical reagent costs and incomplete mineralization, which produces persistent transformation products.

Non-thermal cold plasma has emerged as a unique and mechanistically superior AOP because, unlike all established oxidation methods, it simultaneously generates multiple reactive species, including ˙OH, ozone (O_3_), H_2_O_2_, peroxynitrite (ONOO^−^), singlet oxygen (^1^O_2_), solvated electrons (e_aq_^−^) and UV photons at atmospheric pressure and near-ambient temperatures without the addition of chemical reagents.^15^ Cold plasma can simultaneously oxidize parent antibiotic compounds, attack transformation by-products and inactivate microorganisms and their resistance determinants in a single treatment setup. Among the available plasma sources, dielectric barrier discharge (DBD) has emerged as the most practical configuration for treating antibiotics in wastewater due to its operation at atmospheric pressure, scalability through electrode stacking, and the dense, reproducible micro-discharge arrays that sustain high reactive oxygen and nitrogen species (RONS) yields. DBD systems produce both short-lived ˙OH) and long-lived (H_2_O_2_, O_3_, NO_2_^−^ and NO_3_^−^) RONS, providing oxidative pathways that adapt to diverse antibiotic structural chemistries.

This review focuses specifically on DBD cold plasma applied to antibiotic degradation in wastewater systems. The study aims to establish a connection between plasma physics and antibiotic structural chemistry, addressing a gap in the literature, where the mechanistic understanding of how antibiotic class determines degradation outcomes under DBD conditions remains incomplete. This review is structured to achieve four key objectives: (1) to describe class-specific plasma degradation pathways; (2) to evaluate DBD reactor configurations and the role of individual reactive species in each pathway; (3) to critically assess the reported reduction of ARGs and the inactivation of antibiotic-resistant bacteria (ARB); and (4) to analyze the key process parameters and their influence on RONS yield.

Unlike previous reviews, which broadly survey cold plasma for water treatment or focus on a single antibiotic class, this review introduces a class-dependent plasma antibiotic reactivity framework that explicitly links antibiotic molecular structure to degradation mechanisms and energy requirements under DBD conditions. This framework provides a predictive basis for optimizing plasma treatment parameters according to the target antibiotic class and establishes systematic comparisons that have not been previously presented in the literature. While brief comparisons with other cold plasma configurations are included in Section 3.1 to contextualize DBD performance, this review focuses exclusively on DBD systems for mechanistic analysis, reactor optimization and ARG elimination.

## 2. Conventional methods for antibiotic removal from wastewater

### 2.1 Biological treatment processes

#### 2.1.1 Conventional activated sludge (CAS) technology

CAS works on the principle of aerobic heterotrophic biodegradation, in which a mixed microbial consortium oxidizes dissolved as well as particulate organic matter under controlled hydraulic retention time (HRT) and sludge retention time (SRT).^16^ Such wastewater treatment plants have consistently reported antibiotic removal efficiencies ranging from 10% to 60%, with their performance depending on the antibiotic class present in the wastewater, influent concentration, HRT and SRT (Table 1).^17^ β-Lactam antibiotics are relatively labile to hydrolysis and are typically the most efficiently removed, whereas tetracyclines, fluoroquinolones and macrolides, which are characterized by strong sorption to sludge particles and low biodegradability, tend to remain largely recalcitrant, typically showing removal rates below 30%.^18^ Along with limited degradation, CAS also does not mitigate the spread of ARGs. The high microbial density and accumulation of organic matter in the sludge matrix provide an optimum environment for ARGs and facilitate horizontal gene transfer (HGT) among bacterial populations, which eventually amplifies resistance.^19^ Unlike CAS, DBD plasma can combine parent antibiotic degradation with a reduction in resistance gene abundance, and can selectively oxidize both the parent compound and its transformation products, a combination of activities that CAS cannot perform.

**Table 1:** Comparative performance of conventional technologies for antibiotic removal from wastewater^a^

S. no.	Treatment method	Target antibiotic	Removal efficiency	Concentration	Treatment time	Reference
1	Adsorption	Ciprofloxacin, sulfamethoxazole	61–71%	210 mg L^−1^, 550 mg L^−1^	200 min	44
2	Activated sludge WWTP	Ciprofloxacin, sulfamethoxazole, tetracycline, trimethoprim	95.5%, 93.2%, 98.4%, 76%	10 µg L^−1^ each	3 h	45
3	Anaerobic MBR	Ciprofloxacin	76%	1.5 mg L^−1^	120 days	46
4	Chlorination	Ciprofloxacin, sulfamethoxazole, tetracycline, trimethoprim	97.3%, 96.2%, 97.1%, 96.4%	10 µg L^−1^ each	1 h	45
5	Conventional sewage treatment plant	Sulfamethoxazole	67%	0.58 µg L^−1^	—	47
6	Fixed-film activated sludge MBR	Sulfadiazine, sulfamethoxazole, sulfathiazole	95.1%, 94.3%, 97.8%	150 µg L^−1^	2 months	48
7	Magnetic fullerene nanocomposite	Ciprofloxacin	100%	65 mg L^−1^	153 min	49
8	Multi-walled carbon nanotube filter	Amoxicillin, ciprofloxacin, sulfamethoxazole	98%, 20%, 95%	5 mg L^−1^	60 min	50
9	Multistage slow sand filtration	Trimethoprim	>99%	0.2 mg L^−1^	14 days	51
10	Membrane microalgal-bacterial coupling system	Sulfamethoxazole	39.76%	10 mg L^−1^	135 days	52
11	MBR	Clarithromycin, erythromycin, sulfamethoxazole, sulfamethazine, trimethoprim, roxithromycin	92.3%, 84.2%, 69.7%, 77%, 85.9%, 86.1%	1235 ng L^−1^, 933 ng L^−1^, 301 ng L^−1^, 21.7 ng L^−1^, 35.5 ng L^−1^, 1045 ng L^−1^	40 days	18
12	MBR	Amoxicillin	80%	100 µg L^−1^	170 days	53
13	MBR	Tetracycline	72.79%	2.32 ng L^−1^	—	54
14	Ozonation	Amoxicillin, ciprofloxacin	99%, 96%	100 mg L^−1^	30–120 min	55
15	Photocatalytic ozonation	Ciprofloxacin	92%	20 mg L^−1^	30 min	56
16	Photocatalysis	Cephalexin	94.71%	100 mg L^−1^	100 min	57
17	Photocatalytic degradation	Ciprofloxacin	50%	4 mg L^−1^	60 min	58
18	Reverse osmosis	Oxytetracycline	92%	1000 mg L^−1^	—	59
19	Sub-surface flow constructed wetland	Sulfamethoxazole, trimethoprim	87%, 99%	—	2.5 days	60
20	Trickling filter	Sulfonamides, tetracyclines	23.93%, 23.64%	200 µg L^−1^	120 days	61

a Direct quantitative comparisons across entries should be made with caution. Key experimental parameters are listed in each row to aid interpretation.

#### 2.1.2 MBR system

The MBR system combines a CAS bioreactor with a micro- or ultrafiltration membrane, thereby separating HRT from SRT.^20^ Removal efficiencies of 40–90% have been reported for these systems, which are significantly higher than the 10–60% removal reported for CAS for the same antibiotic class.^21^ The membrane barrier physically traps suspended solids and adsorbed antibiotics, lowering the concentration of β-lactams, fluoroquinolones, tetracyclines and macrolides in the effluent.^22^ Despite this, some of the persistent antibiotics are still able to cross the membrane, mainly those with high hydrophobicity. The capital outlay for membrane modules, pumps and other supporting equipment can be 2 to 3 times that of a comparable CAS plant, while membrane fouling demands frequent cleaning and membrane replacement, as well as higher energy consumption for maintaining transmembrane pressure.^23^ The superior performance comes with high economic and operational costs, making MBR technology impractical for small and medium-scale wastewater plants.^24^

### 2.2 Physical treatment processes

#### 2.2.1 Activated carbon adsorption

Adsorption-based methods, including powdered activated carbon (PAC), granular activated carbon (GAC), zeolite filtration, biochar adsorption and resin-based columns, have been widely tested for their antibiotic removal efficiency in wastewater.^25^ GAC and PAC exploit the aromatic π-electron domains of carbon to adsorb antibiotics through hydrophobic interactions, metal-bridge complexation and π–π stacking interactions for fluoroquinolones and tetracyclines.^26^ Removal efficiencies of 70–99% for antibiotics having aromatic scaffolds, such as ciprofloxacin, norfloxacin and oxytetracycline, have been reported because these molecules bind strongly to the carbon matrix.^27^ The drawbacks are the need for post-treatment and energy-intensive regeneration of carbon (thermal reactivation or chemical washing), as well as the environmental burden associated with carbon production and final disposal. Recent studies have demonstrated that ARGs co-adsorb with drug molecules and may be released during regeneration or when exhausted carbon is disposed of, posing a risk of ARG remobilization in downstream environments.^28^ Activated carbons or zeolites that act as regenerative materials may also exhibit reduced performance after several cycles, which renders the technique unsuitable for efficient use.^29^ Unlike adsorption, which merely transfers antibiotics from the liquid to solid phase without chemical transformation, DBD plasma oxidatively degrades antibiotic molecular structures through radical-mediated bond cleavage. This eliminates the need for adsorbent regeneration, avoids ARG remobilization during disposal, and converts parent compounds into smaller fragments rather than concentrating them on a secondary matrix.

#### 2.2.2 Membrane filtration

Another study showed that nanofiltration (NF) and reverse osmosis (RO) membranes achieve 90–99% removal of most antibiotic classes based on size exclusion and charge repulsion.^30^ The hydraulic pressure required for these processes comes with high energy costs, with operating pressures of ≈5–10 bar for NF and ≈15–30 bar for RO, which translate into specific energy consumption values of 5–20 kWh m^−3^ of treated water.^31^ The process also generates effluent that contains antibiotics at concentrations higher than those in the influent, creating the need for further treatment (advanced oxidation or incineration) or safe disposal. The capital outlay for high-pressure pumps and pretreatment, together with recurring membrane replacement expenses due to fouling and scaling, limits the economic viability of NF and RO.^32,33^ However, DBD plasma technology operates at atmospheric pressure without the high hydraulic pressure (5–30 bar) required for NF/RO, resulting in substantially lower energy costs (0.5–3 kWh m^−3^) while achieving chemical degradation rather than physical removal, thereby eliminating the concentrate disposal problem.

### 2.3 Chemical oxidation methods

#### 2.3.1 Chlorination

Chlorination is a widely employed disinfection technique due to its low cost and high residual disinfection capacity, but its efficacy for antibiotic removal is variable, ranging from 20% to 80% depending on the structure and reactivity of the antibiotic.^34^ Moreover, the oxidizing potential of chlorination is often insufficient to break down more recalcitrant antibiotic compounds, particularly those containing aromatic ring systems such as fluoroquinolones. Another significant drawback is the formation of potentially toxic disinfection by-products (DBPs), such as trihalomethanes (THMs) and haloacetic acids (HAAs), through reactions between chlorine species and natural organic matter (NOM) in source waters. Furthermore, chlorination is not effective at reliably removing ARGs, which continues to pose risks for resistance proliferation in aquatic ecosystems.^35^ Unlike chlorination, DBD plasma produces a wider range of oxidants simultaneously and does not generate the chlorinated DBPs that limit chlorination.^36^ Additionally, plasma-generated RONS directly break DNA strands and oxidize bases in ARGs.

#### 2.3.2 UV disinfection

UV irradiation at 254 nm is effective for some UV-sensitive antibiotics, such as tetracyclines, that have strong absorbance properties at this wavelength range.^37^ However, its application is often impeded in real wastewater because of the presence of turbidity and dissolved organic matter that can scatter or sometimes absorb UV radiation, and therefore impede UV penetration and decrease treatment effectiveness. In the absence of additional treatment steps, UV disinfection generally achieves only partial degradation of parent antibiotics and can generate transformation products that are more persistent than the parent compounds.^38^ Furthermore, UV can cause damage to DNA bases, such as adenine and guanine, that are also found in ARGs, but may still fail to completely inactivate or eliminate such resistance genes. The photolytic mechanism is similar for UV and DBD plasma; however, DBD plasma also yields chemical oxidants (˙OH and O_3_) and can induce direct electron-impact pathways that are not present in UV treatment. This multi-pathway mechanism allows plasma to break apart both UV-transparent and UV-absorbing antibiotics, as well as to oxidize ARGs, simultaneously.

#### 2.3.3 Ozonation

Ozonation is recognized as a highly effective AOP for antibiotic degradation, typically achieving removal rates of 60–95% by directly attacking electron-rich sites within antibiotic molecular structures.^38^ Ozonation of bromide-containing wastewater can lead to the potential formation of bromate, a regulated carcinogen, which poses a serious environmental concern. Furthermore, the high energy consumption required for O_3_ generation (∼10–20 kWh kg O_3_) and the substantial capital investment required for O_3_ generators make ozonation economically challenging.^39^ Ozonation also results in incomplete mineralization of compounds, meaning that the resulting oxidation by-products may persist in the environment or may even exhibit enhanced toxicity compared to the parent antibiotic compounds. However, DBD plasma systems generate O_3_*in situ* alongside ˙OH, H_2_O_2_ and UV, eliminating the need for separate O_3_ generators and their associated energy costs.^36^ The simultaneous presence of multiple reactive species in plasma enables attack on transformation products that resist O_3_ alone and may therefore achieve potentially higher mineralization than ozonation at a lower total energy input, although this has yet to be verified by systematic total organic carbon (TOC) measurements.

#### 2.3.4 Fenton and photo-Fenton processes

The Fenton and photo-Fenton processes are also highly effective AOPs, primarily due to the formation of potent ˙OH from reactions of ferrous ions (Fe^2+^) with H_2_O_2_.^40^ These methods, due to the *in situ* interactions between radicals, can achieve comparably higher removal efficiencies, often 85–99%, mainly because they can oxidize a broad spectrum of organic pollutants.^41^ However, their practical application is limited by the strict requirement for an acidic pH range (typically 2–4) for optimal radical generation. These low pH levels favor the precipitation of large amounts of iron hydroxide in the sludge, which necessitates costly and complex post-treatment disposal procedures.^42^ In contrast, DBD plasma overcomes the fundamental pH limitation of Fenton processes by generating ˙OH through electron-impact water dissociation rather than Fe^2+^/H_2_O_2_ chemistry, enabling effective operation across a broad pH range (3–11) without iron salt addition.^43^ This eliminates iron hydroxide sludge generation, while maintaining ˙OH-mediated degradation efficiency comparable to, or greater than, that of Fenton processes for most antibiotic classes.

### 2.4 Limitations of conventional methods and emerging role of cold plasma

**Fig. 1:**
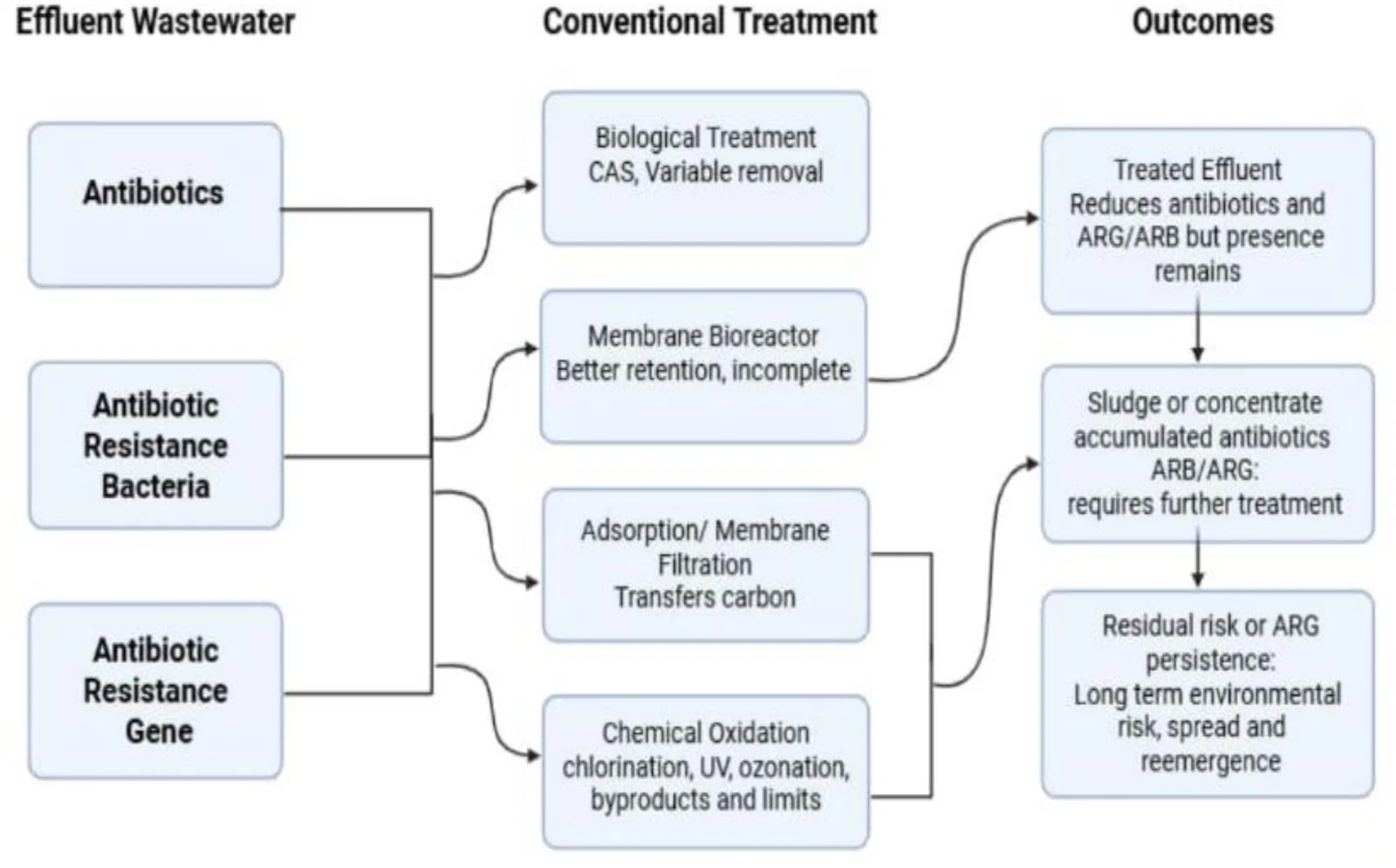
Key limitations of conventional wastewater treatment methods for antibiotic removal.

Conventional treatments remain insufficient for antibiotic-containing wastewater (Fig. 1). Biological systems operate with moderate efficiency in removing antibiotics, and adsorption and membrane processes transfer contaminants to sludge and concentrate them, which still need to be disposed of. Alternatively, AOPs remove pollutants more effectively but may generate toxic intermediates and by-products. Real wastewater presents further challenges, including NOM, salinity and the simultaneous presence of reactive species that inhibit degradation kinetics.^62^ Combined, these limitations demonstrate that no single conventional method can simultaneously achieve complete removal of parent antibiotics, mineralization of the resulting transformation products and control of ARGs, and thus the use of advanced treatments is required.

On the contrary, cold plasma offers a mechanistically broader solution because its near-ambient operation under electrical discharge generates a dense cocktail of RONS, electric fields and UV photons that can oxidize parent compounds, attack intermediates and by-products and inactivate microorganisms in a single step.^63^ Atmospheric cold plasma has been shown to degrade ofloxacin and ciprofloxacin in municipal and industrial wastewater effluents while also reducing their antimicrobial activity. DBD systems have been shown to remove fluoroquinolones from real hospital wastewater, along with reductions in chemical oxygen demand (COD) and ammonia levels.^64^ Cold plasma can achieve rapid removal of the parent antibiotics and, under favorable conditions, partial mineralization of the transformation products. Additionally, cold plasma has the capacity to break down extracellular DNA and inactivate microbial pathogens, which reduces ARG dissemination, even in hospital wastewater matrices where ciprofloxacin removal reached 100% and ARG-related DNA damage was also observed.^65^

## 3. Overview of cold plasma technology

Cold plasma, often described as the fourth state of matter, is a partially ionized gas in which a fraction of the constituent molecules are dissociated, ionized, or excited, while the overall plasma remains neutral.^66^ The collective electromagnetic behavior of plasma allows it to interact over vast distances and produce intricate electrical and chemical changes. Plasma can be classified into two main categories: equilibrium (thermal) and non-equilibrium (non-thermal) plasmas. In thermal (equilibrium) plasmas, species such as ions, radicals, neutrals and electrons share a common temperature, typically reaching several thousand kelvins. Because of their high heat and energy density, these plasmas are commonly utilized in industrial operations, including metal cutting, welding and surface coating.^67^ However, such high temperatures make thermal plasmas inappropriate for heat-sensitive processes, such as wastewater treatment, because of their high energy demand and the risk of thermal destruction of sensitive organic molecules. In contrast, non-thermal or cold plasma maintains a low gas-phase temperature (25 °C) because the electron population is energetically decoupled from the reactive species. The energetic electrons (∼1–20 eV) collide with neutrals, generating reactive species such as ˙OH, O_3_, hydrogen peroxide (H_2_O_2_), and superoxide anions (O_2_^−^), as well as UV photons without appreciable bulk heating.^68^

### 3.1 Foundational plasma physics and mechanism

#### 3.1.1 Discharge generation and micro-discharge formation

Cold plasma is generated by imposing a high electric field across a gas gap, which accelerates electrons in the 1–20 eV range, sufficient to dissociate, ionize and electronically excite gas molecules while leaving the surrounding liquid essentially isothermal. These processes yield reactive species (˙OH, O_3_, H_2_O_2_, peroxynitrite, UV photons and various ions) directly either in the liquid phase or at the gas liquid interface.^69^

DBD is a non-thermal, self-pulsing plasma formed when high-voltage alternating current (AC) is applied across electrodes separated by at least one dielectric layer, commonly made of quartz, glass, alumina, polymers, or ceramics.^70^ The system includes gaps between electrodes of about 0.1 to 10 mm, operating voltages ranging from several kV to 50 kV and frequencies from 100 Hz to a few tens of kHz, with pulsed systems reaching voltages of up to 70 kV and 500 to 2000 Hz.^71,72^ Breakdown starts with a pre-phase exposure that can last several hundred nanoseconds, which then develops into a cathode-directed positive streamer that crosses a 1 mm gap in about 2 ns to form the micro-discharge. The micro-discharge then collapses within 10 ns as surface charge accumulates and covers the field, hence preventing arc formation.^73^ Micro-discharge abundance rises with voltage amplitude, and higher filament density increases the electrode area covered per half-cycle.^74^ Higher dielectric permittivity enhances field distribution and charge deposition, but stability may be reduced, as observed for quartz and alumina *versus* lower permittivity polypropylene (PP) and polycarbonate (PC), which sustained uniform discharge over a broader voltage range in a 2.5 kHz, 2 mm barrier configuration.^72^

#### 3.1.2 RONS generation

In cold plasma, a series of physical, aqueous, plasma-liquid interfacial, gas-phase and biological/genetic processes degrade antibiotics. RONS are generated by energetic electrons and UV light in the discharge. These species then transform into secondary and tertiary RONS, which dissolve into the liquid and attack antibiotic functional groups, break down molecules, and eventually form small inorganic end products. In the sections that follow, each step is thoroughly explained, the reactions are presented and the processes are linked to the results observed for the main classes of antibiotics.

#### 3.1.3 Gas-phase chemistry (production of primary species) and main physical events

High electric fields (DBD, corona, or jet) between electrodes accelerate free electrons, which then collide with molecules of the background gas (air, O_2_, N_2_, and H_2_O vapor). Excited atoms, ions, and species are created by these electron-impact events:^75^

Gas-phase primary electron-impact reactions:e^−^ + O_2_ → O˙ + O˙ + e^−^ (R1: atomic oxygen formation/dissociation)e^−^ + N_2_ → N˙ + N˙ + e^−^ (R2: atomic nitrogen formation)e^−^ + H_2_O → ˙OH + ˙H + e^−^ (R3: hydroxyl radical formation)e^−^ + O_2_ → O_2_^+^ + 2e^−^ (R4: ionization)
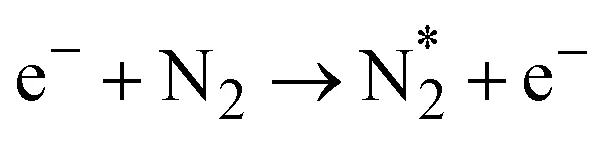


Secondary oxidants are produced *via* three-stage processes and rapid gas-phase recombination of these primary radicals and excited species:O˙ + O_2_ + M → O_3_ + M (R6: ozone formation)˙OH + ˙OH → H_2_O_2_ (R7: hydrogen peroxide)N˙ + O˙ → NO, NO + O → NO_2_ (R8: nitrogen oxides into nitrate chemistry)

The gas-phase emits UV photons (from excited N_2_ and O_2_ species) and creates brief shock/pressure effects that enhance interfacial transport. These main and secondary species provide substrates for liquid-phase chemistry.

#### 3.1.4 Interfacial mass transfer and formation of reactive oxidants in plasma liquid systems

According to Henry's law and mass-transfer principles, reactive gas-phase species transfer into the liquid at the plasma–liquid interface, whereas electron/water interactions directly produce certain species in the near-surface aqueous layer. Important aqueous species that persist long enough to spread and react throughout the bulk include hydrated electrons (e^−^(aq)), O_3_, H_2_O_2_, and NO_2_^−^/NO_3_^−^:^76^

Typical reactions involving interfacial/aqueous formation:O_3_(g) ⇄ O_3_(aq) (R9: gas–liquid partition)˙OH + ˙OH → H_2_O_2_ (R10: preformed in gas-phase or in water)e^−^ (from discharge) + H_2_O → e^−^(aq) + H + ˙OH (R11: solvated electron formation)NO + ˙OH → HNO_2_ → NO_2_^−^/NO_3_^−^ (R12: nitrogen oxide aqueous chemistry)

The tertiary species (O_3_, H_2_O_2_, and NO_*x*_) can produce secondary radicals (such as ˙OH) *in situ* through well-known reactions and have lifespans ranging from seconds to days in water.

#### 3.1.5 Generic radical-mediated reaction steps

Once in solution, antibiotic molecules react with short-lived radicals (˙OH, ˙H, and O˙) and longer-lived oxidants (O_3_ and H_2_O_2_) through a limited number of basic elementary steps: reactions of generic radicals with pollutants (where R stands for the antibiotic moiety) are as follows:R–H + ˙OH → R˙ + H_2_O (R13: hydrogen abstraction/hydroxylation)R + ˙OH → R(˙OH) → ring-opened fragments (R14: addition to aromatic or heteroatoms)R + e^−^(aq) → reduced fragment + X^−^ (R15: reductive dehalogenation/cleavage)R + O_3_ → oxidation products (R16: especially for conjugated/aromatic systems)

### 3.2 Physicochemical properties of key reactive species

#### 3.2.1 ˙OH

˙OH is the principal oxidant in DBD-based water treatment and reacts with other organic compounds near the diffusion-controlled limit. Its second-order rate constants with organic compounds are mostly in the range of 10^8^ to 10^10^ M^−1^ s^−1^. The concentration of ˙OH produced is verified using radical probes, such a *para*-chlorobenzoic acid (*p*CBA) and nitrobenzene, which are reliable^77^ because they track radical-driven oxidation more effectively than direct O_3_ attack. In parallel, EPR spin trapping with 5,5-dimethyl-1-pyrroline N-oxide (DMPO) confirms ˙OH formation through the characteristic 1 : 2 : 2 : 1 quartet of the DMPO-˙OH adduct (a nitroxide radical).^78^

#### 3.2.2 O_3_

O_3_ is more selective than ˙OH and therefore preferentially attacks electron-rich moieties, especially activated aromatics, amines and other nucleophilic sites in antibiotic structures.^79^ In DBD systems, because O_3_ functions as both a direct oxidant and a precursor to secondary radical chemistry, its concentration is best interpreted using the probe data along with matrix effects rather than as an isolated oxidant.^80^

#### 3.2.3 H_2_O_2_

H_2_O_2_ is one of the most important long-lived RONS generated in DBD plasma systems and is a relatively weak direct oxidant compared to the oxidant power of ˙OH radicals in the system (*E*_0_ = 1.78 V for H_2_O_2_*vs.* 2.80 V for ˙OH radicals), although it is a mechanistically significant species in the degradation of antibiotics. DBD-treated water contains enough H_2_O_2_ (10 to 100 mg L^−1^ as a function of the discharge power and/or of the gas composition) to remain present for hours to days and to continue to have an oxidative effect well after the plasma treatment has finished.^81^

The main pathway for H_2_O_2_ to aid in the degradation of antibiotics is its ability to act as a reservoir and a precursor for the generation of secondary ˙OH radicals *via* Fenton chemistry.^82^ In the presence of Fe^2+^ ions, which are usually found in real wastewater or may be released by electron erosion, H_2_O_2_ will undergo the Fenton reaction, as described below:Fe^2+^+ H_2_O_2_ → Fe^3+^ + ˙OH radical + ˙OH^−^

The regeneration of Fe^2+^ from Fe^3+^ can be enhanced by plasma-generated e_aq_ and UV photons. Additionally, H_2_O_2_ participates in UV-driven ˙OH radical generation within the plasma discharge zone:H_2_O_2_ + *hν* (UV, wavelength <280 nm) → 2˙OH radicals.

H_2_O_2_ can also react with O_3_ to generate ˙OH radicals *via* the peroxone pathway:H_2_O_2_ + 2O_3_ → 3O_2_ + ˙OH radicals

These secondary radical generation pathways make H_2_O_2_ a productive intermediate rather than a terminal oxidant, extending the effective treatment duration beyond the plasma exposure period and contributing to the post-discharge degradation of antibiotic residues and transformation products.

#### 3.2.4 Plasma UV

There is also a distinct photolytic pathway with plasma UV, especially in the 200–280 nm range, where many pharmaceutical chromophores, such as antibiotics, absorb strongly. This radiation can directly degrade the structure of the antibiotics that absorb UV rays, and in the case of DBD water treatment systems, it can also enhance the indirect oxidation process by breaking H_2_O_2_ or related intermediates, particularly for aromatic or conjugated antibiotics.^83^

#### 3.2.5 RNS

In air-fed or humid DBD systems, RNS such as NO· and ONOO^−^ become increasingly important because nitrogen oxides dissolve directly into the liquid, where they are converted to nitrous and nitric acid species, while peroxynitrite is generated at the gas–liquid interface.^84^ Together, these species carry out nitration pathways that matter most in real wastewater matrices containing nitrogenous solutes. They can also generate nitro products or alter aromatic compounds like non-steroidal anti-inflammatory drugs (NSAIDs) through covalent modification rather than simple oxygenation.^85^

### 3.3 Application of cold plasma to antibiotic wastewater treatment

The oxidative power of reactive species formed in cold plasma allows it to cleave aromatic rings, dealkylate side chains and, under favourable conditions, mineralize persistent antibiotics to inorganic end products. Cold plasma, without the need for additional chemical reagents or high temperatures, was shown to remove cefuroxime and ciprofloxacin from actual hospital effluent by over 99% while also lowering COD and ammonia.^86^ Because the solution temperature remains low, this technology is well suited to water treatment applications where heat-labile contaminants and microbial communities must be preserved. A classification of cold plasma generation technologies and their characteristics for wastewater applications are shown in Table 2.

**Table 2:** Classification of cold plasma generation technologies and their discharge mechanisms for antibiotic removal from wastewater

S. no.	Plasma source type	Discharge mechanism	Primary RONs	Target antibiotics	Sample	Working gas	Gas flow rate	Sample volume	Time	Removal efficiency	References
1	Atmospheric plasma jet	High-voltage pulsed discharge between inner high-voltage hollow electrode and outer grounded annular electrode; plasma jet exits capillary and interacts with the gas–liquid interface	˙OH, ^1^O_2_, H_2_O_2_, ONOOH	Ampicillin	Ultrapure water	O_2_	20 L min^−1^	200 mL	16 min	94%	87
2	Atmospheric plasma jet	Plasma plume directly injected into liquid, interaction mode: gas–liquid plasma (jet + bubbling/plume contact)	˙OH, ^1^O_2_, O_3_, ONOO^−^	Chloramphenicol, norfloxacin	Ultrapure water	O_2_	20 L min^−1^	—	16 min	Chloramphenicol: 74.60%, norfloxacin: 82.96%	88
3	Atmospheric plasma bubbles	Gas-liquid plasma *via* microbubble generation	˙OH, O_2_^−^, ^1^O_2_	Cefixime	Synthetic water	Air	2.0 SLM	50 mL	30 min	94.8%	89
4	Atmospheric cold plasma	High-voltage plasma generated in an air atmosphere. Plasma interacts with the liquid surface (gas–liquid interface)	˙OH → O_3_, H_2_O_2_, O, HO_2_, NO, NO_*x*_, ONOOH	Ciprofloxacin, ofloxacin	Aqueous solution	Air	—	25 mL	25 min	Ciprofloxacin: 89%, ofloxacin: 92%	90
5	Atmospheric pressure plasma jet coupled with peracetic acid	Plasma jet injected into liquid (gas–liquid interaction)	˙OH, CH_3_, ^1^O, ONOO^−^, O_2_^−^ and NO	Amoxicillin	Real wastewater	Air	8.6 m s^−1^	1 L	2 min	64%	91
6	Aeration-assisted non-thermal plasma	Glow high-pressure discharge; gas-phase plasma transferred into liquid *via* bubbling	˙OH, O_3_, H_2_O_2_	Sulfamethazine, sulfathiazole, sulfamethoxazole	Slaughterhouse wastewater	Air	5 L min^−1^	1 L	—	100%, 100%, 100%	92
7	Contact glow discharge electrolysis	High-voltage DC applied between anode and cathode in liquid; plasma forms at electrode–liquid interface (anode region)	˙OH, H_2_O_2_, HO_2_	Amoxicillin	Distilled water	No external gas	—	300 mL	30 min	97.63%	93
8	Co-axial dielectric/plasma bubble reactor	Air bubbling corona/plasma-catalyst discharge, interface: gas–liquid interface (bubble-induced plasma region)	˙OH, O_2_, O_3_, H_2_O_2_, N_2_, N_2_^+^, UV photons	Tetracycline	Antibiotic-contaminated aqueous wastewater	Air	1 L min^−1^	250 mL	24 min	85.1%	94
9	DBD plasma reactor	Parallel plate (gas–liquid interface), plasma generated above the liquid surface	˙OH, O_3_, ONOOH	Levofloxacin, sulfadiazine	Ultrapure water	Air, O_2_, N_2_, He	1 L min^−1^	40 mL	10 min	Levofloxacin: 89.07%, sulfadiazine: 95.29%	95
10	DBD plasma reactor	Gas-phase pulsed DBD, surface discharge at gas–liquid interface	˙OH, H_2_O_2_, O_3_	Oxytetracycline	Simulated wastewater	Air	1 L min^−1^	900 mL	20 min	93.4%	96
11	DBD plasma reactor	High-voltage AC applied between central rod electrode and outer electrode; gas-phase plasma generated inside quartz tube; reactive species transported into liquid *via* gas diffuser (bubbling system)	˙OH, H_2_O_2_	Pefloxacin	Simulated aqueous solution	O_2_, air	0.125 L min^−1^	100 mL	15 min	97%	97
12	DBD plasma reactor	Plasma is generated in the gas-phase inside a quartz cylinder; a thin liquid film forms along the inner wall; plasma interacts directly with the liquid	˙OH, ^1^O_2_, O_2_^−^	Enrofloxacin	Simulated wastewater	Air	0.3 L min^−1^	300 mL	18 min	98%	98
13	DBD plasma reactor	Atmospheric-pressure DBD, operates in filamentary micro-discharge regime	N_2_(C–B), N_2_^+^, O_2_^+^/O, ˙OH, NO_*γ*_, Cu/Cu^+^	Rifampicin	Deionized water	N_2_, O_2_, air	50 mL min^−1^	25 mL	80 min	74%	99
14	Gliding arc discharge plasma-coupled photocatalysis	Arc ignition at minimum electrode gap (3 mm); arc glides upward due to gas flow	˙OH, NO·, NO_*x*_, H_2_O_2_, H_2_O, O_2_, N_2_	Levofloxacin	Ultrapure water	Humid air	2.5 L min^−1^	250 mL	85 min	100%	100
15	Multi-micro-hole bubble plasma reactor	Underwater bubble plasma generated in gas–liquid interfacial micro-discharges	˙OH, O_3_, H_2_O_2_, O_2_,^1^O_2_	Norfloxacin	Synthetic wastewater	Air	1 SLM	200 mL	50 min	42.6%	101
16	Nanosecond pulsed gas–liquid DBD plasma reactor	Plane–to–plane configuration, atmospheric non-thermal plasma at gas–liquid interface	O, O_2_^+^, ˙OH, O_3_, N_2_, N_2_^+^, NO, NO_2_, NO_*x*_, HNO_2_, HNO_3_	Enrofloxacin	Ultra-pure deionized water	Air, O_2_, N_2_	1.0 L min^−1^	8.5 mL	20 min	100%	102
17	Non-thermal arc discharge pencil plasma jet	Breakdown of humid air → formation of plasma channel; electrode erosion contributes Cu/Cu^+^ species	˙OH, NO N_2_, N_2_^+^ O˙, Cu, Cu^+^	Methotrexate	Synthetic pharmaceutical wastewater	Air	15 L min^−1^	1000 mL	9 min	100%	103
18	Pulsed corona discharge plasma	High-voltage pulsed discharge between needle electrodes (HV) and plate electrode (ground); plasma generated in gas–liquid phase with bubbling air	˙OH, O_3_, H_2_O_2_, O, O_2_^−^, H	Tetracycline	Simulated aqueous solution	Air	6 L min^−1^	300 mL	60 min	82.64%	104
19	Pulsed corona streamer discharge plasma	Gas-phase discharge interacting with liquid surface	˙OH, O, ^1^O_2_, O_3_ O_2_^−^, HO_2_	Amoxicillin	Distilled water	O_2_	300 mL min^−1^	330 mL	60 min	93%	105
20	Pulsed discharge plasma	Gas-phase discharge interacting with liquid surface; high-voltage pulsed plasma generates energetic electrons (e^−^)	˙OH, O_3_, H_2_O_2_	Chloramphenicol	Deionized water	Air	4 L min^−1^	—	60 min	98.4%	106
21	Surface discharge plasma	Plasma generated along dielectric surface	˙OH, O_3_, H_2_O_2_, ^1^O_2_, O_2_^−^	Ciprofloxacin, sulfadiazine, tetracycline	Synthetic wastewater	Air	2.5 L min^−1^	500 mL	20 min	Ciprofloxacin: 58.5%, sulfadiazine: 81.2%, tetracycline: 93.3%	107
22	Submerged cold plasma discharge	Liquid-phase plasma with electrodes immersed in wastewater	˙OH, H, O_2_^−^˙, O_3_, H_2_O_2_	Amoxicillin, ciprofloxacin, cefuroxime, ofloxacin	Real hospital wastewater	Air	4 L min^−1^	600 mL	30 min	Amoxicillin: 75.80%, ciprofloxacin: 99.20% cefuroxime: 99.60%, ofloxacin: 72.13%	108

#### 3.3.1 Types of cold plasma sources for liquid treatment

Cold plasma technology can be classified according to the geometry of the discharge and how plasma contacts the aqueous phase. The most frequently employed configurations for antibiotic remediation in wastewater are DBD, atmospheric pressure plasma jet and low-pressure glow discharge systems. Their performance is primarily assessed based on four key criteria that directly impact wastewater treatment practice involving the yield of RONS, energy efficiency (amount of RONS generated per unit of electrical energy), scalability to pilot or full-scale applications and practical implementation (reactor complexity, materials compatibility and ease of integration).

#### 3.3.2 DBD cold plasma reactor systems

In DBD, plasma is generated between two electrodes separated by a dielectric barrier. The discharge occurs at atmospheric pressure and can be operated in either a planar, coaxial, or packed-bed geometry (Table 3).^109^ Because of the high electric field, electrons accelerate to energies of 1–20 eV, while the bulk gas remains near ambient temperature. DBD produces a dense mixture of long-lived (H_2_O_2_, O_3_, NO_2_^−^ and NO_3_^−^) and short-lived (˙OH) RONS. The atmospheric pressure system eliminates the need for vacuum equipment, thus facilitating scale-up by simply stacking electrode plates or by extending the discharge length in a cylindrical reactor.^80^ Energy inputs range from 0.5 to 3 kWh m^−3^ for >90% antibiotic removal, corresponding to a relatively high RONS-to-energy ratio when optimized for uniform micro-discharges.^110^ DBD reactors are robust and require only standard high-voltage supplies, which can be integrated directly with an immersed flow or liquid film configuration, making them suitable for a continuous-flow systems.

**Table 3:** DBD reactor configurations for antibiotic wastewater treatment^a^

Sr. no.	DBD reactor configuration	Working gas	Antibiotic class	Compound name	Time	Removal efficiency	References
1	Atmospheric air cold plasma	Air	Fluoroquinolone	Ofloxacin, ciprofloxacin	25 min	Ofloxacin: 92%, ciprofloxacin: 89%	90
2	Atmospheric-pressure DBD plasma reactor	O_2_	Sulfonamide	Streptozotocin, trimethoprim, sulfamethoxazole	60 min	90% of each	125
3	Atmospheric air DBD reactor with water falling film	Air	β-Lactams, cephalosporin	Cefixime	8 min	100%	126
4	Atmospheric pressure cold plasma jet reactor	Air	Amphenicols, fluoroquinolone	Norfloxacin, chloramphenicol	16 min	Norfloxacin: 80.06%, chloramphenicol: 70.61%	88
5	Atmospheric air-pulsed DBD plasma reactor	Compressed air	Nitroimidazole	Metronidazole	30 min	93.5%	127
6	Atmospheric air-pulsed DBD plasma reactor	Compressed air	Nitroimidazole	Metronidazole	30 min	93.5%	127
7	Bubbling corona discharge needle–plate reactor	Air	Sulfonamide	Sulfamethoxazole	60 min	94.2%	128
8	Coaxial immersed DBD plasma reactor	Air	Nitroimidazole	Metronidazole	90 min	60.71%	129
9	Coaxial DBD microbubble reactor	Air	Cephalosporin	Cephalexin	20 min	75%	130
10	Coaxial DBD reactor	Air	Tetracycline	Tetracycline	30 min	92.30%	131
11	Coaxial cylindrical DBD plasma reactor	Ar	Macrolide	Erythromycin	60 min	90%	132
12	Coaxial DBD plasma bubble reactor	Air, Ar, O_2_	Sulfonamide	Sulfamethoxazole	20 min	99%	133
13	Circulating-water electrode DBD with double aeration	Air	Fluoroquinolone	Ciprofloxacin	8 min	98.20%	134
14	Cold atmospheric plasma jet	Air, O_2_	Antibiotic mixture	Chloramphenicol, norfloxacin	15 min	Chloramphenicol: 82.89% (air plasma), 91.85% (O_2_ plasma), norfloxacin: 93.95% (air plasma), 93.23% (O_2_ plasma)	88
15	Cold plasma + MWCNT hybrid reactor	O_2_	Fluoroquinolone	Ciprofloxacin, ofloxacin, levofloxacin	60 min	Ciprofloxacin: 90.6% ofloxacin: 83%, levofloxacin: 72.4%	135
16	DBD/UV	—	Fluoroquinolone	Enrofloxacin	30 min	93.90%	136
17	DBD plasma reactor	He, O_2_	Fluoroquinolone, sulfonamide	Levofloxacin and sulfadiazine	10 min	Levofloxacin: 89.07%, sulfadiazine: 95.29%	95
18	DBD reactor coupled with ZNO and persulfate	Air	Fluoroquinolone	Ofloxacin	20 min	With ZNO: 82.8%, with persulfate: 82.8%	137
19	DBD plasma jet reactor	Air, O_2_	Sulfonamides	Sulfamethoxazole	Air: 30 min, O_2_: 5 min	Air: 98.48%, O_2_: 99.50%	138
20	DBD cold plasma reactor	Air, O_2_	Sulfonamide	Sulfamethoxazole	20 min	99.82%	139
21	DBD and peroxymonosulfate	Air	Sulfonamide	Sulfadiazine	15 min	91.8%	140
22	DBD non-thermal plasma	—	Amphenicols	Chloramphenicol	60 min	70%	141
23	Double DBD plasma bubbling reactor	Air	Fluoroquinolone, tetracycline	Norfloxacin, tetracycline	40 min	97.4%, 100%	142
24	DBD plasma jet	He	β-Lactams	Amoxicillin	10 min	90%	143
25	Floating electrode Di-DBD reactor	Air	β-Lactams	Ampicillin	5 min	100%	144
26	Falling-film DBD reactor	Air	Sulfonamide	Sulfadiazine	∼15 min	96%	145
27	Falling-film DBD reactor	Air	Amphenicols	Chloramphenicol	140 min	88.70%	146
28	Falling-film DBD reactor	Air	Fluoroquinolone	Enrofloxacin	60 min	94.35%	147
29	Gliding arc non-thermal plasma reactor	Air	Tetracycline	Tetracycline	30 min	67%	148
30	Gas-liquid nano-pulsed DBD reactor	Air	Fluoroquinolone	Enrofloxacin	20 min	100%	102
31	Gas-phase DBD plasma reactor	Air	Tetracycline	Oxytetracycline	20 min	93.4%	96
32	Gliding pulsed DBD reactor	Air	Tetracycline	Oxytetracycline	20 min	93.6%	96
32	Immersed multi-electrode DBD reactor	Air	Nitroimidazole	Metronidazole	30 min	93.50%	127
33	Nano-second DBD plasma bubble reactor	Air, Ar, N_2_	Tetracycline	Tetracycline hydrochloride	10 min	90.81%	149
34	Non-thermal plasma jet reactor	Air, Ar, N_2_, O_2_	β-Lactams	Amoxicillin	N_2_: 5–7 min, air: 7–10 min, Ar: 10 min, O_2_: 10 min	N_2_: 100%, air: 100%, Ar: 98%, O_2_: 88%	150
35	Nanosecond-pulsed cold atmospheric plasma	Air, N_2_, O_2_	β-Lactams, cephalosporin	Cephalexin, cephazolin	20 min	99.00%	151
36	Nanosecond pulsed DBD wet-wall reactor	Air	Nitroimidazole	Metronidazole	25 min	92%	152
37	Nano-pulsed coaxial DBD plasma bubble reactor	Air, Ar, O_2_	Diaminopyrimidine	Trimethoprim	5 min	83.2%	153
38	Non-thermal pencil plasma jet	Air	β-Lactams	Ampicillin, cloxacillin	9 min	Ampicillin: 76.10%, cloxacillin: 99.99%	154
39	Nano-pulsed pin-to-plate cold plasma discharge reactor	Air, N_2_, O_2_	Cephalosporin	Cephalexin, cefazolin	20 min	Cephalexin: 99.3%, cefazolin: 96.1%	151
40	Plasma DBD reactor	Ar	Macrolide	Roxithromycin, spiramycin, josamycin	30 and 60 min	Roxithromycin, 33.8%, 58.1%, spiramycin: 38.1%, 70%, josamycin: 46.1%, 70%	155
41	Packed-bed DBD cold plasma reactor	Air, Ar, O_2_	Fluoroquinolone	Norfloxacin	8 min	98%	156
42	Parallel-plate DBD reactor	Air, He, N_2_, O_2_	Fluoroquinolone, sulfonamide	Levofloxacin, sulfadiazine	10 min	Levofloxacin: 89.07%, sulfadiazine: 95.29%	95
43	Parallel plate non-thermal plasma reactor	Air	Lincosamide	Clindamycin phosphate	15 min	100%	157
44	Pulsed corona discharge	Air	Glycopeptide	Vancomycin	2 h	90%	158
45	Surface DBD cold plasma reactor	Air	Tetracycline	Lincomycin, oxytetracycline	60, 90 min	Lincomycin 60%, oxytetracycline 100%	159
46	Surface discharge plasma	—	Fluoroquinolone, sulfonamide, tetracycline	Ciprofloxacin, sulfadiazine, tetracycline	20 min	Ciprofloxacin: 58.5%, sulfadiazine: 81.2%, tetracycline: 93.3%	107
47	Trickle-bed DBD reactor	Air	Tetracycline	Tetracycline hydrochloride	60 min	86%	160
48	Uniaxial wet-wall DBD reactor	Air	Fluoroquinolone	Enrofloxacin	18 min	94.6%	98
49	Wet-wall circulating DBD reactor coupled with Ag_3_PO_4_ activated carbon fibers	Air	Fluoroquinolone	Levofloxacin	18 min	63%	161

a Direct quantitative comparisons across entries should be made with caution. Key experimental parameters are listed in each row to aid interpretation.

Several studies have further demonstrated the versatility of DBD reactors for antibiotic degradation across different configurations. In one study,^95^ a DBD system was employed for the simultaneous removal of levofloxacin and sulfadiazine, achieving >90% degradation within 30 min. Another study^96^ evaluated a gas-phase DBD reactor for oxytetracycline removal, demonstrating the influence of gas composition on degradation kinetics. A study^97^ reported effective pefloxacin removal using DBD plasma, with a detailed elucidation of the degradation pathways. Another study^98^ demonstrated the simultaneous removal of enrofloxacin and Cr(vi) using a uniaxial wet-well DBD configuration, highlighting the potential for multi-contaminant treatment (Table 2).

#### 3.3.3 Atmospheric pressure plasma jet reactors

Air plasma jets are high-frequency-powered systems consisting of inner electrodes surrounded by a grounded outer tube, generating a collimated plasma plume that is either immersed in a liquid or diffused into it through a gas phase stream.^111^ Rapid production of ˙OH, O_3_, H_2_O_2_ and ONOO^−^ is possible due to the high local electron densities generated, which depend on the geometry of the jets. The overall RONS yield per unit of power is significantly lower than that of a planar DBD since the active volume is limited to the cross-sectional area of the plume. Therefore, for the treatment of a larger volume of water, multiple parallel jets or a recirculating batch flow design is required. The energy efficiencies reported for antibiotic degradation are generally in the range of 0.8–2.5 kWh m^−3^, explicitly indicating that more energy is required to maintain the plume compared to that required for degradation reactions.^112^ In practice, the composition of the gas (typically He, Ar, or a mixture of N_2_/Ar and/or Ar/O_2_) and careful management of the heat loads at the nozzle can become limiting factors for long-term operation in industrial applications.

#### 3.3.4 Glow-discharge (low-pressure) systems

Glow discharges are sustained in a uniform electric field at low pressures (0.1–10 Pa) and generate a highly homogeneous plasmas with atomic oxygen and excited nitrogen species. The low pressure stops quenching caused by collisions and allows very high concentrations of species (·O, O(^1^D), and N(^2^D)) to be generated per unit of input energy.^113^ However, the need for vacuum pumps and sealed reactors significantly limits scalability and increases capital costs. Typical energy consumption values in most laboratory studies are between 5 and 10 kWh m^−3^ for efficient antibiotic removal. Other implementation concerns include the use of compatible materials to maintain vacuum seals and the requirement for degassing at the downstream end to prevent the discharge of radicals in the gas phase into the treated water.^34^

DBD offers several advantages over the other configurations: the electrode geometry can be scaled, the RONS density is high and the system operates at atmospheric pressure. Each configuration nevertheless retains specific strengths: atmospheric-pressure plasma jets offer a very high density of radicals per unit volume, which is beneficial for treating small volumes, whereas glow discharge systems produce highly homogeneous plasmas with good species selectivity.^114^ For continuous flow wastewater treatment, the optimal plasma source is the DBD plasma source because of its scalability and robustness, while for point-of-use or small-scale applications, the atmospheric-pressure plasma jet could be preferred. The energy efficiencies (DBD: 0.5–3 kWh m^−3^; atmospheric-pressure plasma jet: 0.8–2.5 kWh m^−3^; and glow discharge: 5–10 kWh m^−3^) need to be considered in the context of the volume processed per time.

### 3.4 DBD reactor configurations for antibiotic wastewater treatment

#### 3.4.1 Coaxial DBD reactors

**Fig. 2:**
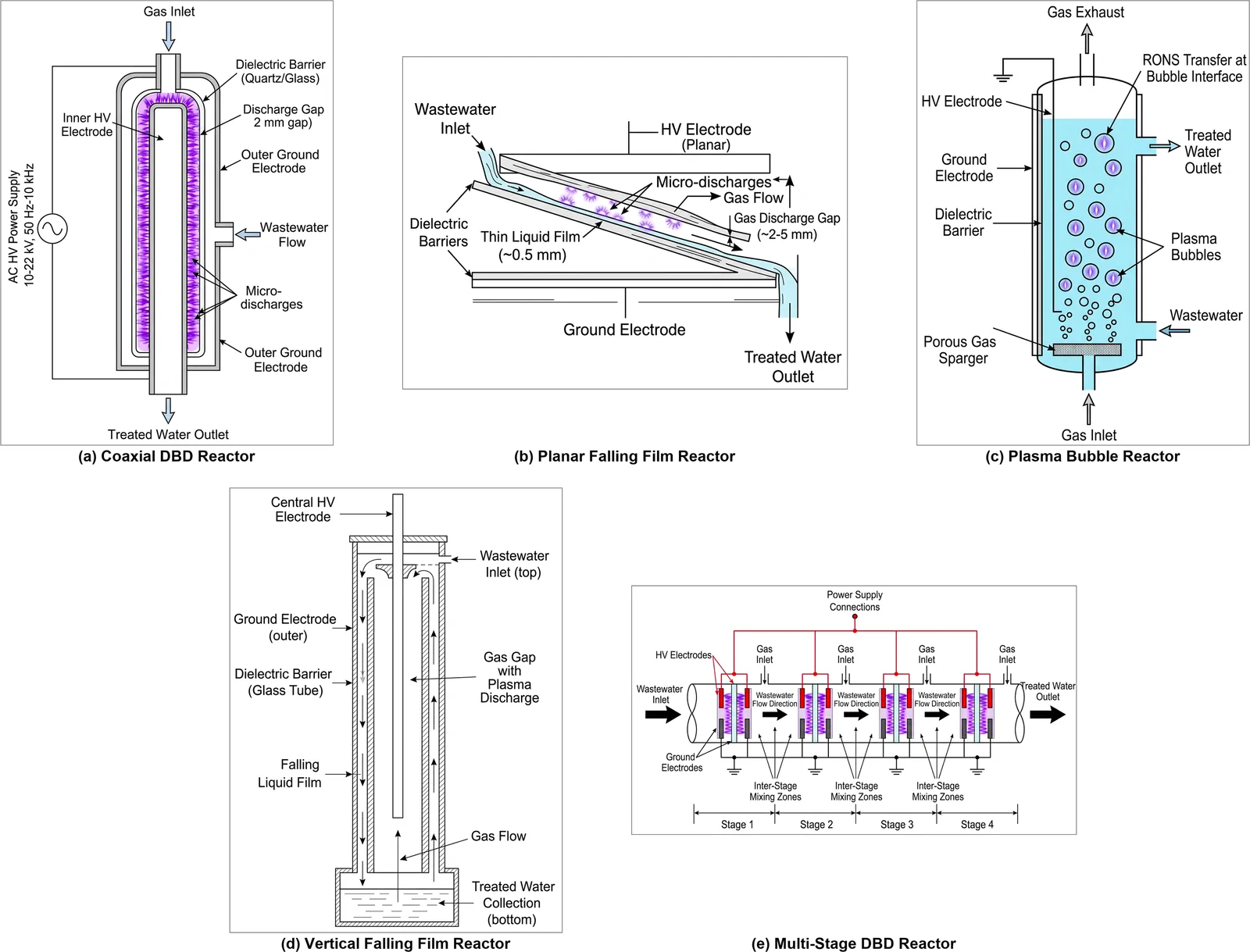
Schematic representation of different DBD reactor configurations for antibiotic wastewater treatment: (a) coaxial DBD reactor; (b) planar falling-film DBD reactor; (c) plasma bubble reactor; (d) vertical falling-film reactor; and (e) multi-stage DBD reactor.

A coaxial DBD reactor (Fig. 2) has one of the most straightforward geometries for wastewater treatment because the discharge is confined to an annular air gap between the dielectric tubes.^115^ The localized active zone is tightly coupled to the sample in the inner tube or the surrounding phase. The inner and outer barriers are usually made of quartz, with diameters of about 1 mm and 7 mm, respectively.^116^ This concentrates the micro-discharges and promotes the transfer of O_3_, UV and short-lived radicals into the aqueous phase, though the treated volume is comparatively limited due to the small discharge cross-section.^80^

#### 3.4.2 Planar and planar falling-film reactors

A planar and planar falling-film reactor (Fig. 2) works by surface renewal through the movement of the liquid as a thin layer under or across the discharge zone.^117^ The short-lived oxidants generated in the plasma react with water before they decay. This configuration is especially efficient for antibiotics such as ibuprofen and diclofenac, as it shortens the diffusion distances that short-lived RONS must travel^118^ and supports faster apparent kinetics than bulk contact systems.

#### 3.4.3 Plasma bubble reactors

A plasma bubble reactor (Fig. 2) uses porous media for sparging, along with a controlled air gap, to disperse feed gas as fine bubbles through the liquid, thereby increasing the gas–liquid interfacial contact area and extending the residence time of gaseous precursors^119^ such as O_3_ and other excited oxygen species. The primary plasma-reactive species formed in these systems are circulated from the gas-phase into the wastewater (liquid phase), where secondary H_2_O_2_ and ˙OH are generated, making the design particularly effective when direct interfacial transfer is the limiting step.^120^

#### 3.4.4 Falling film reactors

Falling-film reactors (Fig. 2) are highly efficient because the liquid film is very thin, continuously renewed and exposed to a large surface area, which maximizes RONS uptake.^121^ Antibiotics such as amoxicillin are degraded with a half-life of less than 1 min and an energy yield of 7213.23 g kW^−1^ h^−1^, showing that film thickness and hydrodynamics can dominate overall efficiency.^122^

#### 3.4.5 Multi-stage DBD reactors

The multi-stage DBD layout (Fig. 2) is better regarded as a process-intensification strategy than as a single geometry and should be presented as a system with the primary stage optimized for RONS generation and a downstream stage optimized for RONS transfer or polishing.^123^ This staged logic is consistent with the broader design principles of DBD reactors, where separating RONS generation, dissolution and post-discharge chemistry improves stability, reduces electrode stress and supports scale-up when a single reactor cannot simultaneously maximize treatment volume and energy efficiency.^124^

## 4. Class-dependent antibiotic degradation

**Fig. 3:**
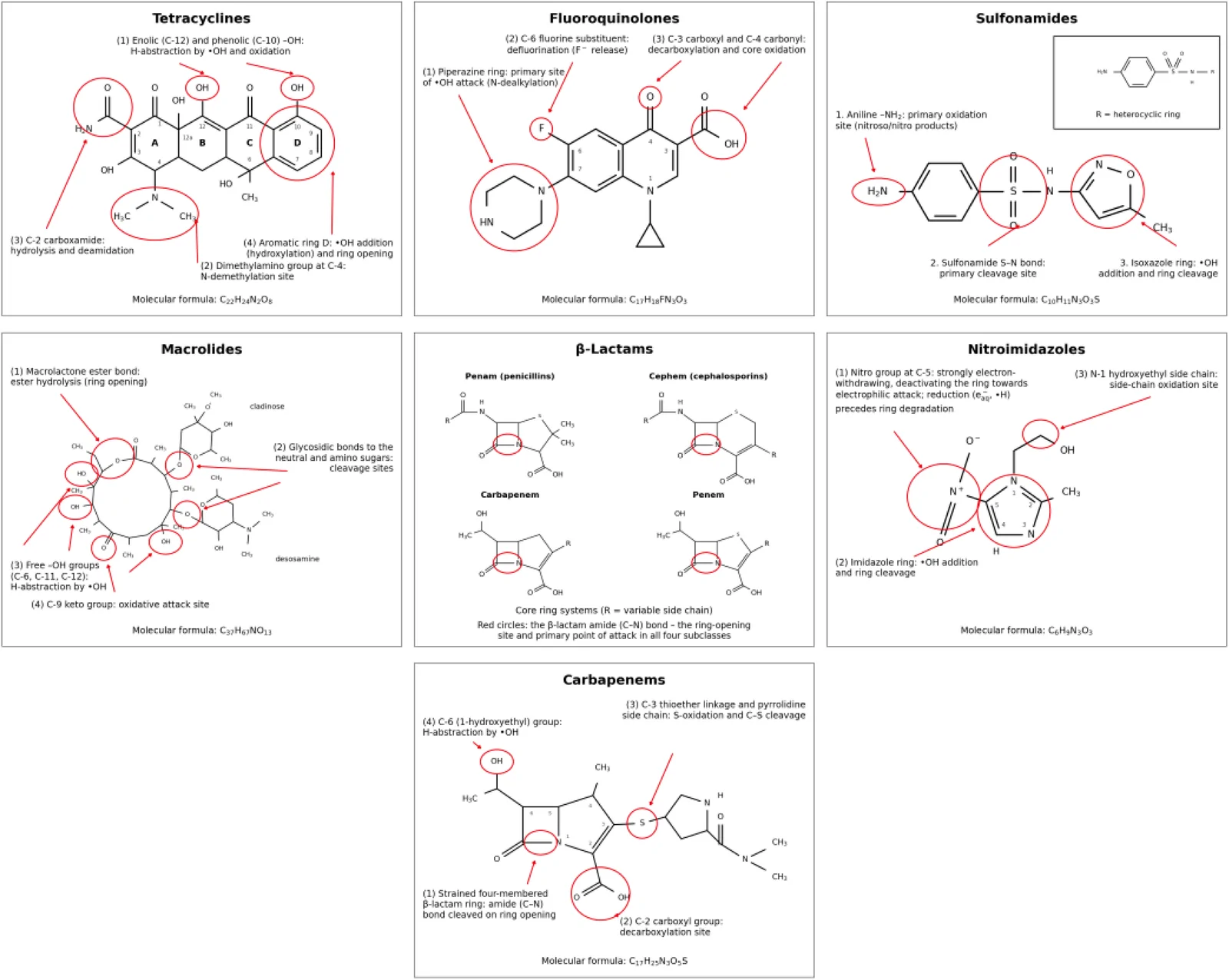
Chemical structure of antibiotic classes and their plasma reactive sites.

Antibiotics have some structural features that determine their sensitivity to plasma-generated reactive species (Fig. 3). The specific oxidation, reduction and cleavage reactions that occur during plasma treatment are determined by variations in aromaticity, ring strain, halogen substitution, and functional side chains. The following are the most prevalent degradation pathways for the most common classes of antibiotics, with particular attention to key reaction steps and experimental observations. The rate constants and removal efficiencies quoted in this section were obtained from studies that used different reactor geometries, initial concentrations, energy inputs and water matrices. They are therefore comparative observations reported in the literature rather than intrinsic constants of antibiotic classes and are best used to rank the relative susceptibility of structural motifs rather than to predict absolute performance.

### 4.1 Tetracyclines

Tetracyclines feature a rigid naphthalene backbone with numerous conjugated double bonds, phenolic hydroxyls, enolic diketones and amide functionality, enabling these molecules to interact easily with plasma-generated oxidants such as ˙OH, O_3_ and O_2_^−^.^162^ The removal rate of tetracycline is 90–97% within 30–60 min under optimized conditions, and degradation is initiated primarily by ˙OH attack on hydroxyl, carbonyl and amino sites, leading to rapid fragmentation.^163^ This same structural complexity that facilitates initial attack also leads to the production of oxygen-containing by-products that prevent complete mineralization,^125^ and this effect is further aggravated by the complexation of divalent cations such as Ca^2+^ and Mg^2+^ with the tetracycline structure. The metal chelation phenomenon is not well investigated but can be significant, especially when these ions are present in real wastewater at concentrations that could affect plasma performance.^164^ Therefore, non-thermal plasma can achieve high removal efficiencies for tetracyclines, but the process usually stops at partially decomposed intermediate products, requiring optimization of plasma parameters and/or the combined use of non-thermal plasma and other AOPs to achieve complete degradation to CO_2_ and H_2_O.^165^

The literature on tetracycline degradation is reviewed and compared critically, showing significant variability among different studies, which is not contradictory but rather can be attributed to differences in the methods used. One study^131^ reported removing over 95% of tetracycline within 30 min in a coaxial DBD reactor treating 50 mg L^−1^ in deionized water, while another study^149^ was able to remove 97% using a plasma bubble reactor treating 20 mg L^−1^ at a lower voltage. This apparent contradiction is mainly due to two reasons: (i) reactor geometry: a 3–5 times higher gas–liquid interfacial area in bubble reactors compared to coaxial systems facilitates more efficient mass transfer of RONS and therefore reduces the voltage level needed for the same removal of tetracycline; and (ii) initial concentration: 50 mg L^−1^ tetracycline consumes more radicals than 20 mg L^−1^, resulting in a lower voltage level required for the removal of 20 mg L^−1^ tetracycline. Most importantly, both studies were not conducted at environmentally relevant concentrations (ng L^−1^ to µg L^−1^) and both used deionized water matrices, which lack the dissolved organic matter that scavenges radicals in real wastewater. Studies performed in real wastewater matrices indicate 30–50% lower removal efficiencies under the same plasma conditions than those reported in studies using synthetic wastewater matrices, as a result of competitive radical consumption by matrix organics. In addition, there is some variability in the reporting of energy efficiency metrics, such as total input power and energy per order (EE/O), which makes it difficult to compare studies. These differences illustrate why the removal efficiencies compiled in this review should be interpreted as condition-specific observations rather than constant values for the tetracycline class.

#### 4.1.1 Mechanism

Tetracycline (C_22_H_24_N_2_O_8_) undergoes rapid oxidation in a DBD cold plasma reactor, where ˙OH and O_3_ serve as dominant oxidants. Initial attack targets the electron-rich aromatic and enolic moieties of the naphthalene scaffold, leading to hydroxylation of the conjugated ring.^165^Tetracycline (C_22_H_24_N_2_O_8_) + ˙OH → hydroxytetracycline/polyhydroxylated intermediates

Oxidative demethylation at the C4 dimethylamino substituent generates secondary amines and hydroxymethyl radicals.–N(CH_3_) + ˙OH → –NH_2_ + ˙CH_2_OH

Continuous oxidative stress then cleaves C=C and C–C bonds within the polycyclic framework and fragments the molecule into low-molecular-weight carboxylic acids, mainly oxalic and formic acids.C=C–C=O + ˙OH/O_3_ → HCOOH (COOH)_2_

However, these acids, being comparatively recalcitrant, accumulate as persistent transformation products, accounting for the observed disparity between the rapid disappearance of the parent tetracycline and the much slower removal of TOC. The identified transformation pathways include hydroxylation, deamidation, demethylation and dehydration, and the resulting intermediates reportedly showed lower acute toxicity than the parent compound.^163^ The predominance of partially oxidized acids explains why parent compound disappearance is rapid, whereas total mineralization remains limited. Mineralization should therefore be claimed for this class only when TOC removal or inorganic end products have been measured.

### 4.2 Fluoroquinolones

Fluoroquinolones have a bicyclic aromatic quinolone core with a fluorine atom on the C-6 position and a piperazine substituent at C-7, which contribute to their high lipophilicity and broad-spectrum activity. This arrangement makes the piperazine ring susceptible to oxidation, while fluorine stabilizes the quinolone ring.^166^

In a DBD cold-plasma reactor, the discharge simultaneously generates a suite of oxidative and reductive agents, including ˙OH, O_3,_ e_aq_ and UV photons, which together attack the piperazine nitrogen, leading to ring cleavage and defluorination.^107^ The intense electric field and micro-discharges at the gas–liquid interface promote water radiolysis primarily through O_3_ and ˙OH, while high-energy electrons injected into the liquid-phase give rise to e_aq_^−^, enabling the reductive cleavage of C–F bonds and dehalogenation of the quinolone ring.^167^ The UV component of the plasma further excites the aromatic rings and facilitates photolytic pathways that complement radical attack.^168^ This synergistic oxidative-reductive environment accelerates hydroxylation of the quinolone core, opening of the piperazine ring and fragmentation of the parent fluoroquinolone into low-molecular-weight compounds; complete mineralization is rarely demonstrated because TOC removal typically lags behind parent compound removal.^169^

The wide range of reported fluoroquinolone removal efficiencies (70–98%) across DBD studies can be attributed to three principal factors.^170^ First, at equal energy input, ciprofloxacin is removed more efficiently in a falling-film DBD reactor than in a coaxial system because the thin liquid film reduces the diffusion path length for the short-lived ˙OH radicals. Second, some studies report removal without clearly distinguishing antibiotic removal in the presence or absence of radical-enhancing additives, such as persulfate or peroxymonosulfate, with removal shifting from ∼60% (plasma alone) to >97% (plasma + persulfate) for the same treatment time. Third, the C–F bond dissociation energy (*ca.* 485 kJ mol^−1^) renders defluorination the rate-determining step, and studies reporting high overall removal may achieve only partial defluorination of the fluoro compound, leaving intermediates that are also fluorinated and thus have unknown ecotoxicity. Additionally, metal ion interference (Zn^2+^ and Cu^2+^) from real wastewater can reduce removal by 15–40% through complex formation with the quinolone moiety.^107^ Future work should report defluorination efficiency alongside parent-compound removal and conduct experiments in representative wastewater matrices. The range of values reported for this class therefore reflects differences in reactor design, additive use and matrix composition as much as differences in intrinsic reactivity.

#### 4.2.1 Mechanism

In a DBD cold-plasma reactor, the initial attack on fluoroquinolones is an electrophilic attack mediated by ˙OH and O_3_, preferentially occurring at the electron-rich quinolone nucleus and generating monohydroxylated intermediates (*e.g.*, hydroxylated ciprofloxacin).^171^Flouroquinolone + ˙OH/O_3_ → hydroxylated intermediate

In a highly oxidizing plasma environment, subsequent radical attack cleaves the piperazine side chain, releasing volatile by-products such as dealkylated amines and formaldehyde.Fluoroquinolone-piperazine + ˙OH → secondary amines + HCHO

Simultaneously, plasma-induced e_aq_^−^ generates a reducing microzone that promotes reductive defluorination, consequently cleaving the robust C–F bond and eventually destabilizing the aromatic system.^172^Fluoroquinolone–F + e_aq_^−^ → carbon-centered radical + F^−^

The loss of fluorine facilitates subsequent oxidative ring opening, yielding short-chain dicarboxylic acids (oxalic and formic acids), which are further mineralized to CO_2_ and H_2_O under sustained plasma exposure.Organic acids + ˙OH/O_3_ → CO_2_ + H_2_O

This cascade of synergistic oxidative-reductive steps explains the rapid degradation of the parent fluoroquinolone, while only partial mineralization is observed without additional treatment stages. Removal efficiencies for norfloxacin typically range from 70% to 90% after 15 min of treatment.^88^ However, the high bond-dissociation energy of the C–F bond renders the degradation of this class of antibiotics more energy intensive than that of non-fluorinated antibiotics.^173^

### 4.3 Sulfonamide

Sulfonamide antibiotics are the third most frequently investigated class of antibiotics, mainly because of their intensive use in veterinary medicine and resulting high loads in agricultural runoff and livestock waste streams.^174^ Sulfamethoxazole is the dominant compound in laboratory and pilot-scale advanced oxidation studies, while sulfamethazine and sulfadiazine, also prevalent in the environment, receive less attention in plasma-based studies. Sulfonamide antibiotics have a (–SO_2_–NH–) linkage that couples an aromatic moiety (isoxazole in sulfamethoxazole, pyrimidine in sulfadiazine, or dimethoxypyrimidine in sulfadimethoxine) to another heterocyclic or aniline fragment. This bond is notoriously recalcitrant to biological degradation yet highly susceptible to oxidative attack by plasma-generated RONS.^175^

It has been found that sulfonamide degradation is critically dependent on the composition of the water matrix, which has not been sufficiently emphasized in the literature when comparing sulfonamide degradation studies. In a nanosecond-pulsed bubble reactor,^176^ an exceptionally low EE/O of 0.29 kWh m^−3^ per order was achieved for sulfamethoxazole, compared to an EE/O of 6–10 kWh m^−3^ per order for the same compound in a conventional DBD system. This is not a fundamental difference in chemistry but primarily attributed to a difference in reactor design, because nanosecond pulses generate a greater population of energetic electrons per unit of input energy. However, the degradation efficiency of sulfonamide is reduced by 40–60% in the presence of humic acid (10 mg L^−1^), representative of real wastewater, because of radical scavenging.^177^ Across studies, this matrix effect is seldom quantified. In addition, the transient toxicity of the intermediates is higher than that of the parent compound during the first 10–20 min of treatment; however, this transient toxicity peak is not mentioned in most of the studies, although prolonged treatment has been demonstrated to restore full detoxification.^178^ There is still a large knowledge gap regarding real wastewater treatment with mixed contaminants, as well as a lack of long-term post-treatment monitoring of intermediate persistence and the ecological effects of treatment.

#### 4.3.1 Mechanism

The isoxazole heterocycle increases the electron density on the sulfonamide nitrogen, thus facilitating electrophilic radical attack. Initial plasma exposure cleaves the S–N bond and converts the sulfonyl fragment to sulfonic acid. Simultaneously, the aniline-derived ring undergoes ˙OH-mediated hydroxylation and O_3_-induced ring opening.^179^ Kinetic studies have reported >90% removal of the parent compound within 20 min of plasma exposure, accompanied by extensive mineralization (∼90% TOC removal) under optimal discharge power and acidic conditions (pH 3–5).^178^ Transient accumulation of aromatic amine intermediates increases the toxicity of the treated solution, while prolonged treatment has been reported to reduce this toxicity in the applied bioassays; detoxification should be claimed only where it is supported by ecotoxicological testing.

Reactive species formed initially through plasma exposure induce S–N bond scission. This step rapidly causes cleavage of the pharmacologically active structure.Sulfonamide + ˙OH → sulfonic acid derivative + isoxazole-containing amine

The aniline-derived aromatic ring undergoes hydroxylation by ROS.Aromatic fragments + ˙OH → hydroxylated intermediates

This hydroxylation weakens the aromatic structure and promotes further oxidation.^180^ Lastly, ring opening is mediated by O_3_, and final mineralization of the resulting intermediates occurs.
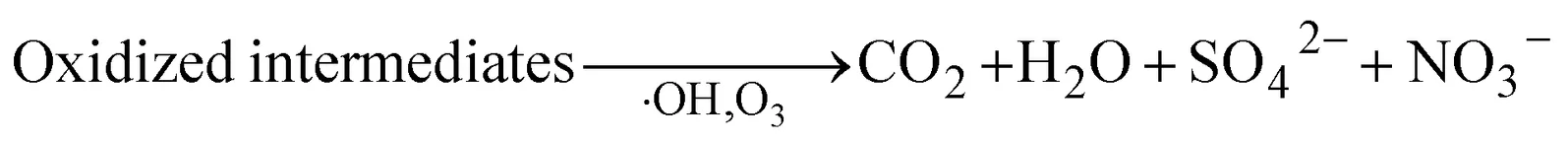


### 4.4 Macrolides

Macrolides are classified as high-priority and critically important antibiotics by the WHO, alongside polymyxins and glycopeptides, because they are essential human medicines with a high risk of resistance and are also among the most frequently detected antibiotics in surface waters and wastewater systems, with erythromycin, azithromycin and clarithromycin repeatedly reported.^181^ They are structurally important because of their large 12- to 16-membered macrolactone ring, which is coupled with multiple hydroxyl and ketone groups and amino sugars connected through ether linkages, all of which are potential reactive sites for plasma-generated oxidants.^182^ Macrolides are major selective agents for the emergence of macrolide-resistant *Streptococci* and are widely used as first-line agents, with resistance in *Mycoplasma pneumoniae* increasing markedly.

The macrolide degradation literature under DBD conditions is notably sparse compared to tetracyclines, fluoroquinolones and sulfonamides,^183^ making it difficult to establish reliable kinetic parameters. The limited studies reported treatment times of 30–60 min for removal of 80–90% of erythromycin at mg L^−1^ concentrations in synthetic water matrices only.^132^ To the best of our knowledge, no study has been conducted to evaluate macrolide degradation by DBD in real wastewater, and the effect of dissolved organic matter, variation in pH and competing pharmaceuticals on macrolide-specific degradation kinetics is completely unknown. In addition, macrolides have a high molecular weight (733–749 Da) and a complex three-dimensional structure, which results in several possible transformation products, but complete identification of these transformation products has not been done under DBD conditions. This is an essential investigation and an important research gap that needs to be filled, since these antibiotics are some of the most commonly found in surface waters in Europe and are part of the EU Watch List.

#### 4.4.1 Mechanism

In DBD cold-plasma systems, macrolide degradation is initiated by oxidation of the dimethylamino group on the desosamine sugar. ˙OH and O_3_ induce N-demethylation or N-oxidation, forming N-hydroxylated intermediates and methyl radicals:Macrolide-N(CH_3_)_2_ + ˙OH/O_3_ → macrolide-N(CH_3_)(˙OH) + ˙CH_3_

The resulting destabilization facilitates radical-driven cleavage of the macrolactone ring through oxidative or hydrolytic pathways:^132^Macrolide lactone ring + ˙OH/O_3_ → open-chain polyhydroxylated intermediates

Glycosidic linkages are cleaved to produce amino sugars and polyols, which are partially oxidized to aldehydes and subsequently to short-chain oxygenated compounds and small amounts of CO_2_.^155^ Standard residence times for DBD systems are insufficient for complete mineralization. The degradation of erythromycin is more likely to occur than that of azithromycin in all reported plasma reactors, which is attributed to differences in their structures (ring size and heteroatom substitution). Macrolide removal is generally more time-consuming or may require a catalytic process because steric hindrance and macrocyclic rigidity limit radical access to the reactive sites. These features account for the slower degradation and the lower extent of mineralization of macrolides relative to β-lactams and tetracyclines.

### 4.5 β-Lactams

The antibiotic class most susceptible to degradation is the β-lactams, because of the four-membered lactam ring, which is highly strained and the carbonyl of the amide is highly electrophilic.^184^ Their degradation in DBD cold-plasma reactors begins mainly through electrophilic attack by ˙OH and O_3_ on the amide carbonyl of the β-lactam ring. This reaction results in a rapid rate of ring opening, leading to the loss of antibacterial activity, which is typical of β-lactams having the fastest plasma degradation kinetics of any antibiotic class.

However, the clinical and environmental significance of this finding needs to be interpreted with caution, as the reported >90% removal efficiencies may reflect a combination of plasma-mediated oxidation and abiotic hydrolysis.^185^ These contributions cannot be separated from studies without proper control experiments (plasma off, same temperature and pH). Moreover, the literature on β-lactam antibiotics is focused on ampicillin and cefixime, whereas DBD studies of clinically important carbapenems (meropenem and imipenem) and cephalosporins (ceftriaxone and cefotaxime) remain scarce. Cephalosporins contain a sulfur-bearing dihydrothiazine ring, which yields sulfur-containing metabolites that have not yet been identified in environmental samples. The data on energy efficiency for the degradation of β-lactams are not consistent, and some studies report total power consumption, whereas others report energy yield (g kW^−1^ h^−1^), making meaningful cross-study comparison difficult. Future studies should include hydrolysis controls, extend coverage to the clinically important subclasses of β-lactams and adopt standardized energy-reporting metrics.

#### 4.5.1 Mechanism

Under DBD cold plasma, β-lactam degradation is initiated by highly reactive species such as ˙OH and O_3_, which preferentially attack the strained β-lactam carbonyl, causing rapid ring opening and loss of antibacterial activity:β-Lactam + ˙OH/O_3_ → cephalosporanic acid

The unstable cephalosporanic acid then undergoes oxidative cleavage of the dihydrothiazine ring and methoxyimino side chain, accompanied by decarboxylation and deamination:^186^Cephalosporanic acid + ˙OH → open-chain intermediates + CO_2_ + NH_4_^+^

Sulfur-containing fragments released from the dihydrothiazine ring are further oxidized to inorganic sulfate:S-containing intermediates + ˙OH/O_3_ → SO_4_^2−^

Finally, low-molecular-weight organic acids (*e.g.*, formic and acetic acids) produced during fragmentation are further mineralized under sustained plasma exposure:^187^HCOOH, CH_3_COOH + ˙OH/O_3_ → CO_2_ + H_2_O

### 4.6 Nitroimidazole

Metronidazole is a widely prescribed nitroimidazole drug for anaerobic infections, such as *Clostridium*-associated diseases and giardiasis, and has become an emerging contaminant in surface and wastewater.^188^ Nitroimidazoles contain a five-membered imidazole ring bearing a strong electron-withdrawing nitro group (–NO_2_), which makes the imidazole ring electron-deficient and thus reduces its susceptibility to electrophilic attack by ˙OH. The primary oxidative step therefore does not involve direct ˙OH attack on the aromatic ring; rather, the nitro group must first undergo reductive activation *via* electron transfer, which then permits oxidative ring cleavage to proceed.^189^

Metronidazole is removed from plasma in a nanosecond-pulsed DBD reactor by metronidazole hydroxylation, which reaches approx. 60% after 60 min of plasma exposure at neutral pH, with hydroxylation being the predominant pathway. The pseudo-first-order rate constants for metronidazole (*k* ≈ 0.02–0.04 min^−1^) are lower than those for the fluoroquinolones (*k* ≈ 0.06–0.12 min^−1^) and tetracyclines (*k* ≈ 0.08 min^−1^), reflecting inherent resistance due to the nitro group.^152^ The influence of gas composition appears to be greater for this class than for others, but is not well documented in the literature because most studies have used air or O_2_ without systematic comparison.

Coupling DBD plasma with sodium persulfate (PS) effectively increases the degradation of metronidazole to >85% *via* sulfate radicals (SO_4_˙^−^) at neutral pH within 60 min, a 25–30% improvement over plasma-only treatment,^129^ though this improvement comes at the cost of added chemical reagents, which diminishes one of the key advantages of plasma over conventional AOPs. Dissolved organic carbon, such as humic acid, present in real wastewater (municipal and agricultural runoff), acts as a scavenger of both ˙OH and SO_4_^−^ radicals; metronidazole removal efficiency drops by 50–70% in the presence of 10 mg L^−1^ humic acid under the same DBD/PS conditions.^190^ This raises serious concerns about the applicability of this process to real wastewater.

#### 4.6.1 Mechanism

Nitroimidazole antibiotics (metronidazole and ornidazole) degrade through a two-stage process where first reduction of the nitro group takes place, followed by oxidative breakdown of the imidazole ring. This happens because plasma generates both reducing species (e_aq_) and oxidizing species (˙OH and O_3_) simultaneously.^191^

The activation of the nitro group (–NO_2_) takes place as it accepts an electron:R–Im–NO_2_ + e_aq_^−^ → R–Im–NO_2_˙^−^

This unstable intermediate quickly undergoes stepwise conversion into:R–Im–NO_2_ → R–Im–NO → R–Im–NHOH(nitro → nitroso → hydroxylamine)

˙OH then attacks the imidazole ring structure:R–Im–NO_2_ + ˙OH → hydroxylated intermediates

The oxidized ring is cleaved and breaks apart into smaller molecules:Hydroxylated intermediates + ˙OH → open-chain compounds

These fragments are converted into simple acids:Open-chain compounds → formic acid (HCOOH), acetic acid (CH_3_COOH), and NH_4_^+^

All intermediates are further oxidized:Simple acids, NH_4_^+^ + ˙OH/O_3_ → CO_2_ + H_2_O + NO_3_^−^

### 4.7 Carbapenems

Carbapenems (imipenem, meropenem, and ertapenem) constitute the last line of therapy against multidrug-resistant Gram-negative infections. Hospital effluents are hotspots for carbapenem-resistant Enterobacteriaceae (CRE, critical priority 1 pathogens).^192^ Their structure has a β-lactam core fused to a bicyclic ring, which makes them comparatively resistant to direct radical attack, so that degradation proceeds preferentially through hydrolysis rather than radical-mediated oxidation. The amphiphilic nature of polymyxins and the dense hydrogen-bond network of vancomycin similarly promote surface adsorption and radical scavenging, further reducing plasma efficiency for these classes.^193^

This structural resistance contributes to an existing data gap, as degradation kinetics, energy metrics, degradation rates, the toxicity of potential intermediates or by-products, and transformation pathways of carbapenems, polymyxins, glycopeptides and linezolid are not well characterised under DBD plasma conditions, thus limiting the risk assessment of treated effluents. Additionally, hospital wastewater, which is the largest source of environmental carbapenems, contains complex matrices of high organic content that can greatly affect the efficiency of plasma treatment and make the extrapolation of clean-water kinetic data to actual performance challenging.^194^ The occurrence of multiple ARGs, such as carbapenem resistance genes (blaKPC, blaNDM, and blaOXA-48), in hospital effluents makes it urgent to find new technologies that can treat both ARGs and associated drugs. There is a need for immediate research to determine pseudo-first-order rate constants for these poorly studied classes under different DBD configurations, to identify and quantify transformation products by liquid chromatography-high-resolution mass spectrometry (LC-HRMS), and to assess the energy consumption and toxicity of treated water.^195^ The co-occurrence of these drugs and their resistance determinants in hospital effluents creates an urgent need for treatment technologies that can address both simultaneously.

For all the antibiotic classes studied (Table 4), a distinct structure-reactivity hierarchy can be observed, with the 4-membered lactam ring of the β-lactams being the most susceptible to DBD plasma attack, mainly because of the inherent ring strain of the lactam ring and its highly electrophilic carbonyl group, which readily undergoes attack by ˙OH radicals and O_3_. For the sulfonamides (*k* ∼0.05–0.12 min^−1^), the electron-rich S–N bridge is an easily accessible site for oxidative cleavage. The mid-range rate constants (*k* ∼0.04–0.08 per minute) are comparable for tetracyclines and fluoroquinolones, but the degradation pathways are quite different: tetracyclines are degraded by multi-site hydroxylation of the densely functionalized structure of the naphthacene core, which produces persistent degradation intermediates, while the fluoroquinolones must undergo synergistic oxidative (piperazine cleavage) and reductive (C–F bond scission) pathways. The macrolides are relatively recalcitrant, with a *k* ∼0.02–0.05 min^−1^, due to steric hindrance in their 14- to 16-membered macrolactone ring and limited radical access. The nitro group in nitroimidazoles is associated with slower initial reaction rates (*k* ∼0.02–0.04 min^−1^) since the group prevents direct electrophilic attack on the imidazole ring and requires reductive activation by e_aq_ before the ring can undergo oxidative cleavage. The reactivity classification in this class-dependent reactivity framework shows that the optimization of DBD plasma treatment should not only be optimized for reactor conditions but also tailored to the specific structural chemistry of the target class of antibiotic. The rate-constant ranges mentioned above are compiled from studies that differ in reactor configuration, initial concentration, specific energy input, gas composition and water matrix. They therefore describe the relative behaviour reported in the literature under the conditions of each study and do not represent intrinsic kinetic constants of the antibiotic classes: the ordering of structural susceptibility is consistent across the literature, whereas the absolute values are not transferable between systems.

**Table 4:** Summary of class-dependent antibiotic degradation by DBD plasma reactors

Sr. no.	Antibiotic class	Chemical formula	Dominant structural target	Primary RONS	Rate-determining step	Typical *k* (min^−1^)	Key intermediates	Removal (%)	Time (min)	Reference
1	β-Lactams	4-Membered cyclic amide ring	Strained β-lactam ring	˙OH, O_3_	Ring opening	0.10–0.15	Penicilloic acid, SO_4_^2-^	>90	15–30	185
2	Sulfonamides	C_10_H_11_N_3_O_3_S	S–N bridge	˙OH, O_3_	S–N bond scission	0.05–0.12	Sulfanilic acid, aminoisoxazole	>90	20–40	178
3	Tetracyclines	C_22_H_24_N_2_O_8_	Naphthacene core (multi-site)	˙OH, O_3_	Hydroxylation	0.04–0.08	Oxalic/formic acids	90–97	30–60	163
4	Fluoroquinolones	C_17_H_18_FN_3_O_3_	Piperazine ring + C–F bond	˙OH, O_3_, e_aq_	Piperazine cleavage + defluorination	0.04–0.08	Hydroxylated quinolones	70–98	15–60	88
5	Macrolides	C_37_H_67_NO_13_	Macrolactone ring + amino sugars	˙OH, O_3_	Lactone ring opening	0.02–0.05	Amino sugars, polyols	80–90	30–60	132
6	Nitroimidazoles	C_6_H_9_N_3_O_3_	–NO_2_-shielded imidazole	˙OH, e_aq_	Reductive nitro activation	0.02–0.04	Hydroxylamine, nitroso intermediates	∼60	60	152

## 5. ARG elimination and degradation mechanisms

It is important to distinguish four hierarchically related but operationally distinct outcomes of plasma treatment: (i) parent-antibiotic removal: disappearance of the parent pharmaceutical molecule from the solution, which reduces the associated selective pressure and does not by itself demonstrate mineralization or detoxification; (ii) ARB inactivation (loss of bacterial viability as assessed by culture-based methods); (iii) e-ARG degradation (oxidative destruction of free DNA containing resistance genes in the aqueous phase); and (iv) i-ARG degradation (damage to resistance genes within intact or partially compromised bacterial cells). Progressively higher plasma energy doses are needed for these outcomes: the lowest energy doses are needed for antibiotic removal, moderate energy doses for ARB inactivation, and the highest energy doses for i-ARG degradation (∼2.5-fold higher than those required for e-ARG degradation).^196^ Most importantly, one outcome is not necessarily guaranteed by other outcomes: complete antibiotic removal does not necessarily equate to ARG elimination, as e-ARG DNA can remain in wastewater long after the parent antimicrobial molecule has been degraded,^197^ and ARG inactivation does not necessarily mean ARB inactivation, because bacteria may be rendered non-culturable while their resistance genes remain sufficiently intact to participate in horizontal gene transfer.

### 5.1 Scope and significance of ARG contamination in wastewater

ARGs are inheritable genetic materials that provide bacteria with the capacity to survive antibiotic exposure.^198^ Alternatively integrons, especially class 1 integrons (*intI-1*), serve as mobile genetic platforms that mainly capture and express gene cassettes and therefore act as master vectors for HGT in wastewater.^199^ This makes ARG elimination arguably a more pressing and distinct research objective than parent antibiotic removal: persistent naked or cell debris-associated e-ARG DNA can be acquired by environmental bacteria *via* natural transformation, thus reconstituting resistance in sensitive populations and driving the dissemination of multidrug resistance.^197^

The ecologically most relevant ARGs in wastewater treatment plants (WWTPs) include tet genes (tetracycline resistance), qnr genes (fluoroquinolone resistance), sul genes (sulfonamide resistance), mcr-1 (colistin resistance), bla genes (β-lactam resistance) and intI-1 (class 1 integrons).^200^ Activated sludge samples from WWTPs across six continents have shown numerous distinct ARGs spanning 15 different drug classes. β-Lactam resistance genes dominated the resistome (46.5% of the total ARG abundance), followed by glycopeptide resistance (approx. 24.5%) and tetracycline resistance (16.2%). The core resistome, comprising 20 ARGs present in almost every sampled WWTP, collectively accounted for 83.8% of total ARG abundance. Abundance is expressed as the ARG copy number per bacterial cell, with the highest ARG richness observed in Asia compared to other regions.^201^

### 5.2 ARG elimination by DBD cold plasma

Studies specifically quantifying ARG log reduction by DBD cold plasma remain limited but have expanded rapidly since the first report approximately five years ago.^202^ DBD plasma has been shown to reduce ARG abundance through two distinct pathways: first, direct oxidative DNA damage, in which RONS, particularly ˙OH and O_3_, cause DNA strand breakage, crosslinking and base oxidation, thereby inactivating gene function; and second, indirect elimination *via* antibiotic-resistant bacteria (ARB) inactivation, where RONS, charged particles and UV radiation disrupt the cell envelope and kill the host organism carrying i-ARGs.^203^

In a mesoporous plasma reactor system treating multidrug-resistant *Escherichia coli*, reductions in qPCR-measured gene abundance reached 5.71 log for aac(3)-II, 5.46 log for intI-1, and up to 15.19 log for blaTEM-1 under specific optimized conditions (11 kV, 10 min); reductions of 2–6 log are more typical across different reactor configurations and operating parameters.^204^*E. coli* levels determined by 16S rRNA were reduced by approximately 4.7 log after 15 min of exposure, and gene reductions of 5.8 log in tetA, 5.4 log in tetR, 5.3 log in aphA and 5.5 log in tnpA were observed after 30 min, respectively (Table 5). A plasma dose of 1.06 kJ cm^−2^ in wastewater matrices eliminated e-blaTEM-1 and tet genes by 3.26 and 3.14 log_10_ copies per mL, respectively.^203^ Notably, a plasma intensity of just 0.35 kJ cm^−2^ caused a >1 log reduction of e-blaTEM-1, whereas more than 0.88 kJ cm^−2^ was required to achieve the same level of reduction for intracellular targets. In methicillin-resistant *Staphylococcus aureus* (MRSA), a plasma intensity of 0.12 kJ cm^−2^ achieved a 5 log bactericidal reduction but only 0.19 log degradation of the i-mecA gene, which required >0.53 kJ cm^−2^ for a 1 log reduction.^205^

**Table 5:** ARB and AMR gene degradation using non-thermal cold plasma technology^a^

Sr. no.	ARG type	Target bacteria	ARG log reduction	ARB log reduction	References
1	aac(3)-II	—	aac(3)-II: 1.06 log	—	207
2	amp^R^	*E. coli* DH5α	3.4 log	—	208
3	amp^R^, kan^R^, tetA	*E. coli*, MG1655	2.74 log	6.90 log	209
4	aac(3)-II, blaTEM-1, intI-1, tetC, tetW	—	aac(3)-II: 1.32 log, blaTEM-1: intI-1: 2.46 log, tetC: 2.80 log, 1.34 log, tetW: 2.39 log	—	210
5	aac(3)-II, blaTEM-1, intI-1, tetC, tetW	—	aac(3)-II: 1.32 log, blaTEM-1: 1.34 log, intI-1: 2.46 log, tetC: 2.80 log, tetW: 2.39 log	—	210
6	aac(3)-II, blaTEM-1, intI-1, tetC, tetW	Multi-strain-resistant *E. coli*	aac(3)-II: 5.71 log, blaTEM-1: 5.19 log, intI-1: 5.46 log, tetC: 2.28 log, tetW: 2.88 log	—	211
7	e-mecA, i-mecA	MRSA	e-mecA: 2.6 log, i-mecA: 0.8 log	5 log	212
8	e-blaTEM-1, tet gene	*E. coli*	e-blaTEM-1: 3.26 log, tet gene: 3.14 log	3 log CFU mL^−1^	213
9	e-qnrB, e-blaCTX-M, e-sul2, e-intI-1, i-qnrB, i-blaCTX-M, i-sul2, i-int-1	AR *E. coli*	e-qnrB: 1.99 log, e-blaCTX-M: 2.22 log, e-sul2: 2.66 log, e-int-1: 2.80 log, i-qnrB: 2.01 log, i-blaCTX-M: 1.84- log, i-sul2: 2.40 log, i-int1: 2.73 log	97.9%	202
10	intl-1	AR *E. coli*	4.43 log	7.4 log	214
11	aac(3)-II, blaTEM-1, intI-1, tetC, tetW	—	aac(3)-II: 1.6 log, blaTEM-1: 1.5 log, intl-1: 1.5 log, tetC: 1.5 log, tetW: 2.4 log	—	215
12	blaTEM-1, intl-1, sul1, tetA, tetC	AR *E. coli*	blaTEM-1: 7 log, intl-1: 6.5 log, sul1: 5.8 log, *tetA*: 6.3 log, tetC: 5.9 log	7 log	216
13	aac(3)-II, blaTEM-1, intl-1, tetC, tetW	AR *E. coli*	aac(3)-II: 2.2 log, *blaTEM-1*: 1.84 log, intl-1: 2.3 log, tetC: 1.04 log, tetW: 0.61 log	7 log	217
14	aphA, tetA, tnpA, tetR	*E. coli*	aphA: 5.3 log, tetA: 5.8 log, *tnpA*: 5.5 log, tetR: 5.4 log	4.8 log	218

a Direct quantitative comparisons across entries should be made with caution. Key experimental parameters are listed in each row to aid interpretation.

Comparison of ARG elimination studies reveals substantial variability in reported log reductions that reflects differences in experimental design rather than inherent plasma efficacy.^206^ The sources of discrepancy include: (i) ARG target size, as smaller gene fragments (blaTEM-1, 800 bp) are more likely to suffer strand breakage than larger genes (tetW, 1920 bp), simply because of the size-dependent probability of radical encounters; (ii) ARG quantification method, as qPCR may overestimate gene elimination when damage is concentrated within the primer-binding sites, while the functional core of the gene remains intact, and may equally underestimate damage that falls outside the short amplified region; (iii) ARG state, as e-ARG in free solution is more prone to strand breakage than i-ARG protected by cell envelopes because of the higher probability of radical encounters; and (iv) water matrix, as organic matter and cell debris in water quench RONS before they reach their gene targets. This gap highlights the fact that ARG elimination data from clean-water experiments cannot be directly extrapolated to real wastewater applications, and future studies must quantify both culturability-based and molecular-based (qPCR and metagenomics) endpoints simultaneously.

### 5.3 Mechanisms of ARG inactivation by plasma RONS

#### 5.3.1 DNA strand-break induction

**Fig. 4:**
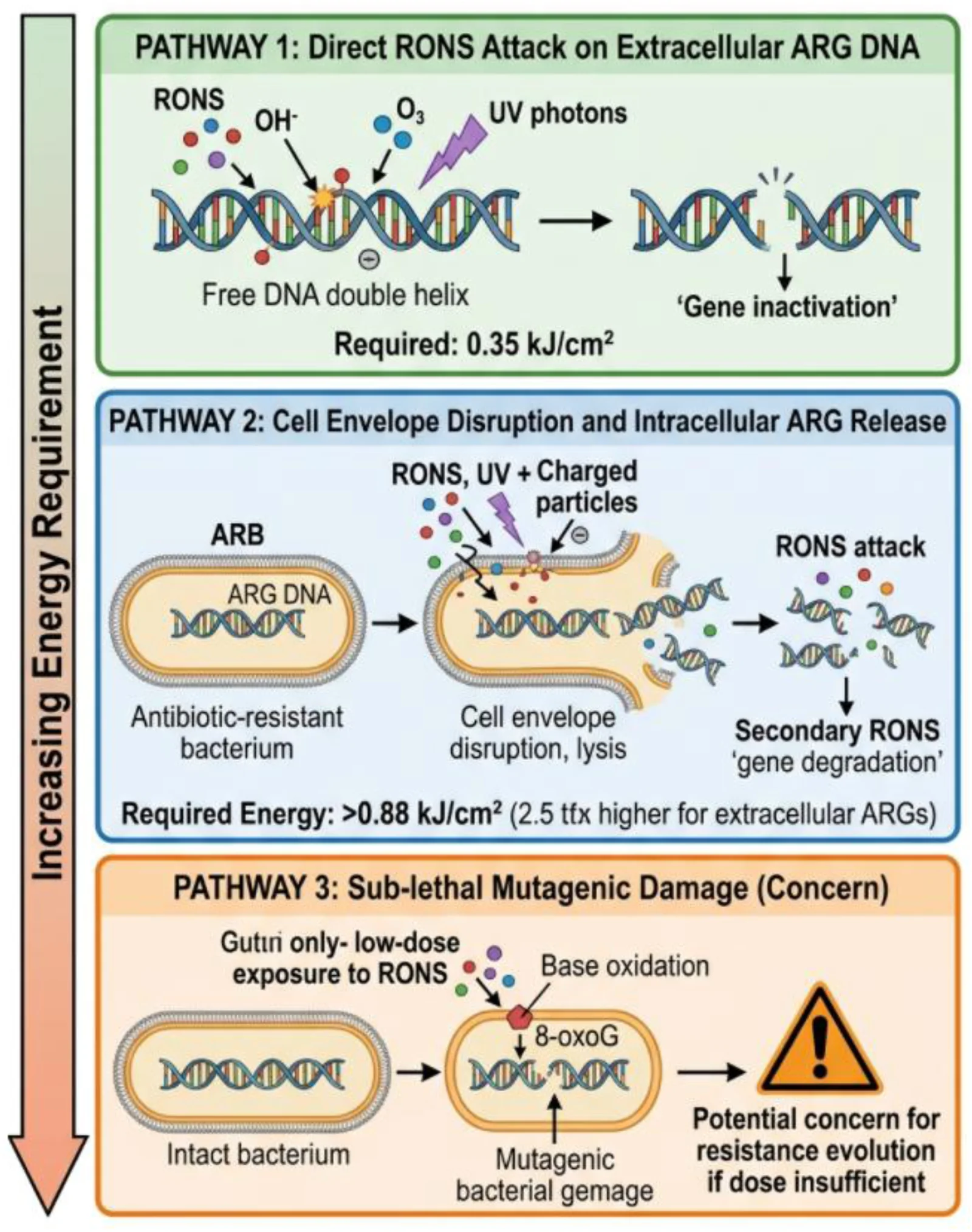
Mechanism of non-thermal cold plasma interaction with antibiotic-contaminated wastewater and simultaneous microbial inactivation of antibiotic-resistant bacteria through cell envelope disruption.

˙OH and O_3_ generated in the DBD plasma system induce both single-strand breaks (SSBs) and double-strand breaks (DSBs) in both chromosomal DNA and plasmid DNA (Fig. 4).^219^ DSBs are non-repairable under normal environmental conditions and hence result in complete inactivation of ARGs. This can be detected using a plasmid supercoiling relaxation assay followed by gel electrophoresis, which provides a direct visualization of this breakage, as parent supercoiled plasmid DNA migrates faster than its linear form.^220^ Longer plasma exposure converts supercoiled bands to open circular and smaller degraded fragments that fall below the detection limit. In a mesoporous plasma system, ARG-containing DNA bands for aac(3)-II, intI-1, bla(TEM-1), tetC and tetW disappeared entirely after 10 min. Single-cell gel electrophoresis (comet essay) has also been used to assess plasma-treated cells, quantify DNA strand breaks, and correlate these breaks with loss of cultivability.^217^

O_3_ preferentially attacks guanine bases and converts them into 8-oxoguanine (8-oxoG), whereas ˙OH directly breaks the deoxyribose and phosphate backbone.^221^ Atomic oxygen reacts with either pyrimidine or purine bases or the deoxyribose backbone, thus forming fragments that are too short to encode functional resistance proteins.^222^

#### 5.3.2 Base oxidation and mutagenic damage

Sub-lethal plasma doses may cause mutagenic effects rather than lethal DNA damage and thus raise an underexplored concern that incomplete treatment may theoretically elevate ARG mutation rates and select for more resilient variants (Fig. 4). The most common and characterized type of oxidative lesion is 8-oxo-7,8-dihydro-2′-deoxyguanosine (8-˙OHdG), which mispairs with adenine and drives G→T transversions.^223^ Mammalian keratinocytes exposed to DBD cold plasma showed an increase in 8-˙OHdG levels from 442 ng mg^−1^ to 1571 ng mg^−1^ after plasma exposure, and this increase was suppressed by antioxidant pretreatment.^224^ Cold-plasma jet irradiation generates 8-oxoG in both intracellular DNA and aqueous DNA, with base mutations detectable at treatment doses too low to induce strand breakage, showing the dose-dependent formation of 8-oxoG and suggesting that low doses can still leave genes structurally intact but functionally altered.^225^

#### 5.3.3 ARB inactivation

Plasma-induced ARB inactivation proceeds through a multi-target assault on the cell envelope, where RONS oxidize membrane lipids, which disrupts barrier integrity (Fig. 4); UV photons cause pyrimidine dimerization and charged particles disrupt membrane potential.^226^ Scanning electron microscopy (SEM) of plasma-treated multidrug-resistant *E. coli* reveals wrinkled, collapsed, and damaged morphologies that progress to complete rupture, causing leakage of ARG-containing DNA into the surrounding medium. Inactivation efficiency is both dose- and species-dependent, as multidrug-resistant *E. coli* is reduced by >3 log in wastewater at 0.71 kJ cm^−2^ and by 6.3 log at 18 kV; MRSA is reduced by > 5 log at 0.12 kJ cm^−2^ in 10 min and carbapenem-resistant *Klebsiella pneumoniae* by 6.92 ± 0.24 log within 60 min of plasma exposure. Non-thermal DBD plasma treatment makes *Staphylococcus aureus* (*S. aureus*) viable but non-culturable (VBNC) at 7.4–7.6 log_10_ CFU mL^−1^ under 8.1–24.3 kJ of applied energy, with upregulation of antioxidant defense genes (katA, msrA, dps, and trxA). *S. aureus* retains its pathogenicity and internalizes into HeLa cells through virulence factors (ClfB, SCIN, SdrD, and SasH) and suppresses host immune recognition, enabling prolonged persistence. The dissociation between ARB inactivation and ARG elimination can be seen in the persistence of vanA genes at ∼10^5^ copies/mL despite >4 log reduction; mecA requires >0.53 kJ cm^−2^ of energy input for 1 log degradation, while only 0.12 kJ cm^−2^ is required for its extracellular counterpart, highlighting that intracellular ARGs are shielded by cytoplasmic components and the cell envelope. These results collectively demonstrate that ARB inactivation is insufficient for ARG elimination and that sub-lethal plasma treatment may only render resistant bacteria undetectable by culture-based methods while preserving their resistance determinants and capacity for HGT.

### 5.4 Degradation mechanisms of antibiotic molecules by DBD plasma

DBD plasma degrades antibiotics through oxidation pathways that depend on discharge conditions, feed gas and the water matrix. In liquid-phase DBD plasma systems, ˙OH is the primary oxidation driver because it is non-selective and chemically reactive, whereas O_3_ and UV photons act as complementary oxidants that enhance the degradation rate. This is not a single reaction route but rather a complex of overlapping processes that convert parent antibiotics into smaller intermediates and, under favorable conditions, ultimately to inorganic products.

#### 5.4.1 ˙OH-mediated hydroxylation pathways

˙OH attack initiates antibiotic degradation by addition to unsaturated aromatic moieties, forming short-lived radical intermediates, which further undergo hydroxylation, successive oxidation and rearrangement.^90^ The intermediates then fragment into lower-molecular-weight products, which also explains why DBD often causes structural loss and produces decolorization even before complete mineralization is achieved. The *R*_ct_ concept is widely used to interpret oxidation performance in ozonation-based AOPs; it expresses the ratio of steady-state ˙OH exposure to O_3_ exposure and has been used as a practical benchmark for mixed ˙OH and O_3_ chemistry.^227^

#### 5.4.2 O_3_-driven electrophilic attack and ring opening

In atmospheric and O_2_-fed DBD systems, O_3_ contributes mainly as a selective oxidant, particularly attacking electron-rich moieties such as C=C bonds and amine-containing groups while simultaneously being used as a precursor for secondary ˙OH through plasma-assisted radical chemistry and aqueous decomposition.^228^ The UV emission from plasma further accelerates direct photolysis of chromophoric antibiotics, while the relative effects of these pathways vary with gas–liquid composition and the operating conditions of reactors. This mechanistic coupling is why DBD performance should be interpreted as a system property rather than the effect of a single oxidant.^151^

#### 5.4.3 Photolysis by plasma UV emission

DBD plasma provides an additional UV-driven pathway that accelerates the antibiotic degradation rate, especially for molecules that contain chromophores with absorbance within the plasma emission window. Such degradation is mostly important in gas-surface configurations where UV photons can directly interact with the liquid interface, but in real-world applications, it usually acts in combination with oxidant chemistry rather than as an isolated mechanism.^229^ Its importance is relative, as it varies with the working gas and reactor geometry, where O_2_-rich systems generally favor oxidant generation and N_2_-containing discharges contribute more to mixed nitrogen chemistry and UV output.

#### 5.4.4 Direct electron impact

Direct electron impact is concentrated at the plasma-liquid interfacial region, where energetic electrons can transiently interact with molecules at or near the surface before rapid thermalization and solvation occur.^230^ This mechanism is most important in systems with a high interfacial area, such as thin-layer reactors or falling-film reactors, whereas submerged and bubble-assisted configurations are dominated by dissolved RONS in the liquid.^231^ Reactor configuration therefore determines whether degradation is controlled by either interfacial electron-driven events or downstream radical chemistry in solution.

#### 5.4.5 Kinetic modeling: pseudo-first-order and Langmuir–Hinshelwood

Most DBD antibiotic studies are interpreted using pseudo-first-order kinetics, which is reasonable only when the target concentration is low enough that the antibiotic does not materially compete for the reactive oxidants.^232^ Once the concentration increases or the matrix becomes more complex, the apparent rate becomes concentration-dependent because the pollutant and background components compete for ˙OH, O_3_ and other plasma-derived species. In catalyst-assisted systems, Langmuir–Hinshelwood behavior is more appropriate because adsorption and surface reactions both contribute to the observed decay profile.^233^ For meaningful comparison across studies, the key requirement is consistent reporting of reactor power, contact time, treated volume and log removal conditions, since these parameters govern the apparent performance as much as chemistry itself.

### 5.5 Transformation products and ecotoxicological considerations

While DBD plasma achieves rapid disappearance of parent antibiotic compounds, the disparity between parent compound removal and TOC reduction is a persistent concern across all studied antibiotic classes.^234^ The removal of tetracycline typically exceeds >90%, whereas TOC removal under the same conditions is generally only 30–40%, suggesting the formation of partially oxidized intermediates such as oxalic, formic and acetic acids. Likewise, treatment with fluoroquinolones produces hydroxylated quinolones and dealkylated amines, which remain in solution. In the case of sulfonamides, transient aromatic amine intermediates (*e.g.*, sulfanilic acid and 3-amino-5-methylisoxazole) are detected and found to have higher acute toxicity than the parent compound in *Vibrio fischeri* luminescence assays before further oxidation of the sulfonamide moiety under extended treatment, which releases sulfate ions that are generally less toxic.

There are limited reports in the literature on the ecotoxicological assessment of plasma-treatment effluents: less than 30% of the published DBD antibiotic reports include some type of toxicity testing and a standardized bioassay protocol is rarely used. Future studies should report TOC or COD removal and transformation-product identification alongside parent-compound removal, and should include multitrophic toxicity testing (bacteria, algae, and crustaceans) of plasma-treated effluents, so that detoxification is demonstrated rather than assumed.

## 6. Influencing parameters and process optimization

A combination of interconnected operational parameters determines the efficacy of DBD plasma for the removal of antibiotics. Although individual parameter optimization schemes for DBD plasma reactors have been widely reported, the field has yet to adopt multivariate optimization frameworks that would better represent the coupled interactions between the plasma and liquid phases. The five main parameters, *i.e.*, applied voltage and input power, discharge frequency and pulse width, working gas composition and flow rate, solution pH and conductivity, and initial antibiotic concentration, are evaluated.

### 6.1 Applied voltage and input power

The first electrical parameter that drives the generation of RONS in DBD systems is the applied voltage. Increased voltage enhances the electric field across the discharge gap, increasing the rate at which electrons are energized to the extent required to dissociate gas-phase molecules (O_2_, N_2_ and H_2_O) and generate a higher density of microdischarges.^235^ This is directly proportional to RONS yield at the gas–liquid interface, thus speeding up the kinetics of antibiotic degradation and accelerating the removal of compounds such as sulfamethoxazole, ciprofloxacin, tetracycline and enrofloxacin.^150^ Ciprofloxacin degradation efficiency increased from 48.90% to over 89.66% with an increase in applied voltage from 165 V to 195 V in 60 min,^236^ and another study reported over 40% improvement in ciprofloxacin degradation when the voltage was raised from 10 kV to 15 kV within 60 min.^237^

The relationship between applied voltage and degradation efficiency is not linear and exhibits a certain voltage threshold, typically around 22 kV for many reactor configurations.^64^ If it exceeds this limit, the effect of dielectric heating and the disproportionate increase in energy consumption start to have a negative effect on the overall efficiency of the system and may cause unwanted side reactions or possibly place strain on the equipment. However, there is still a lack of research on multivariable optimization of voltage along with other variables, such as frequency and gas flow rate, for many antibiotic degradation studies.^238^

Plasma characteristics and generation efficiency of RONS are greatly affected by the type of power supply. A nanosecond-pulsed DBD system generates a higher number of RONS per joule of energy input compared with the traditional sinusoidal excitation power supply system. This is because the pulsed discharge mode enables the production of highly energetic electrons that form high-density populations of reactive species and improve energy coupling into the plasma.^239^

One major drawback in the literature on voltage optimization is that most studies changed the voltage while fixing other parameters, but voltage is a critical parameter for discharge frequency, gas flow rate and gap distance. For instance, the removal at low frequencies (100–500 Hz) increases with increasing voltage (from 10 kV to 22 kV), but at higher frequencies (>5 kHz), increasing the voltage can lead to the formation of arcs and electrode damage, which is seldom studied. Also, it is reported that the optimum voltage is different for different reactor geometries and gap distances, so these values cannot be transferred from one system to another. The field does not have any dimensionless scaling parameters (such as reduced electric field *E*/*N* or specific energy density *J*/*L*) to allow for meaningful cross-reactor comparisons of the effects of voltage. This lack of standardization makes the currently available voltage optimization data reactor-specific and not useful in developing generalizable design guidelines.

### 6.2 Discharge frequency and pulse width

Discharge frequency and pulse width are treated as kinetic control parameters rather than just simple electrical settings. Operating an atmospheric DBD at 200 Hz has been reported to degrade ciprofloxacin completely in soil, and frequencies of 400 to 600 Hz outperform 300 Hz in a nanosecond-pulsed plasma system, which is consistent with the idea that frequency must be high enough for RONS production but not so high that surface charges suppress new micro-discharges.^236^ Pulse width is also important in nanosecond-pulsed systems because it determines the duration for which streamers remain sustained, ranging from roughly 30 to 40 ns, 200 ns, 400 ns, and up to 900 ns, as well as the amount of post-pulse chemistry.^240^

From a methodological point of view, frequency is reactor-specific and must be matched to the reactor's electrical resonance because discharge power, power transfer and thermal behavior can increase up to the resonance point and then deteriorate after that threshold. That is why operating up to the resonant frequency is likely to be the most energy-efficient strategy, whereas frequency mismatch wastes power through reactive loading and may even trigger thermal runaway in pulsed DBD reactors. Hence, optimized operating points such as 200 Hz, 6 kV and 20 kHz or 3 kHz with 2.4 µs pulses, rather than full frequency-to-voltage response variations, are reported.^241^

### 6.3 Working gas composition and flow rate

Working gas composition is a major determinant of plasma chemistry because it determines whether the discharge is dominated by nitrogen-based species or oxidizing ROS. For antibiotic removal in DBD systems, oxygen-rich feed compositions are the most effective because they favor O-atom chemistry, high O_3_ formation and downstream ˙OH production.^80^ On the contrary, atmospheric air (79% N_2_ and 21% O_2_) gives a similar but more diluted oxidizing RONS mixture, while N_2_ or Ar feeds shift the discharge toward NO_*x*_-dominated chemistry. The lower oxygen content inhibits the production of O_3_ and˙OH, while the higher nitrogen content enhances the production of RNS species, such as NO, nitrite (NO_2_^−^), nitrate (NO_3_^−^) and ONOO^−^. Therefore, oxidative efficacy follows the order O_2_ > air > N_2_ > Ar, although this ranking is still reactor-specific and depends on humidity, power and gas–liquid coupling.^242^

This ranking should be interpreted with important caveats. First, it applies to ˙OH-dominated degradation of oxidation-susceptible antibiotics (tetracyclines and sulfonamides) but may not hold for nitroimidazoles, which require reductive activation and may benefit from N_2_-containing feeds that enhance e_aq_ production. Second, a pure O_2_ feed achieves the highest O_3_ yields but incurs significantly higher operational costs than ambient air, and a cost–benefit analysis comparing O_2_ and air feeds at a pilot scale has not been performed for any antibiotic. Third, humidity in the feed gas is a confounding variable that is rarely controlled or reported: water vapor enhances ˙OH production but suppresses O_3_ accumulation, creating a trade-off that shifts the optimal gas composition depending on whether the target antibiotic is more susceptible to ˙OH (tetracyclines) or O_3_ (sulfonamides). These interactions demonstrate that a simplistic gas ranking cannot serve as a universal design criterion and that gas optimization must be performed on a per-antibiotic-class, per-reactor basis, a systematic investigation that is currently absent in the literature.

A DBD system fed with pure O_2_ results in the highest O_3_ concentrations in the gas phase, reaching 2100 ppm at 70 kV and 3200 ppm at 80 kV after 10 min of plasma exposure.^243^ In the case of fluoroquinolone (norfloxacin) degradation, an O_2_ feed ensures high removal since ˙OH is the driving force and O_3_ contributes to secondary oxidation pathways. Air (79% N_2_ and 21% O_2_) gives a mixture of RONS concentrations.^172^

Gas flow rate determines both the residence time of RONS within the discharge zone and the balance of RONS transport to the liquid surface; hence, it must be optimized rather than simply increased. Lab-scale reactors generally operate at around 1 to 2 L min^−1^: below this range, insufficient gas inflow limits the production of RONS, while above this range, decreased residence time prevents adequate energy transfer between gas molecules and electrons, therefore limiting the production of RONS.^244^ An airflow rate of 1 L min^−1^ at 7.5 kV achieved 93.4% oxytetracycline removal in 20 min, showing that moderate flow rates are enough for efficient mass transfer at the lab scale.^245^ For scale-up systems, O_3_ gas emission, capture and destruction require upstream processes and specific design requirements because process efficiency depends on both reactive-species generation and how effectively those species are transferred into the aqueous phase.

### 6.4 Solution pH and conductivity

Solution pH has a multimodal effect on antibiotic degradation in DBD plasma reactors because it affects oxidant lifetimes, antibiotic speciation, and the dominant reaction pathways. Degradation of norfloxacin increases as the system shifts from pH 3 to 7.8 and then to 11, which also increases the electro-ozonation rate constant from 0.007 to 0.022 s^−1^, showing that pH directly alters reactivity through speciation.^246^ pH also controls the oxidant pool: at high pH, a radical chain process involving O_3_ is promoted, increasing indirect ˙OH formation, which strengthens non-selective oxidation and results in O_3_ decomposition^247^ H_2_O_2_ is less stable at higher alkalinity; its decomposition rate is accelerated at near-neutral pH and further accelerated in alkaline media. Near-neutral pH is generally considered an optimal window for plasma systems, since it helps maintain the stability of the oxidants and the activity of the antibiotics to ensure efficient attack by the ˙OH, O_3_ and species derived from H_2_O_2_.^178^

One significant drawback is that the pH may not remain constant throughout the treatment, but instead shift to a new value, thereby altering the oxidant chemistry that an antibiotic will encounter during treatment. The treated water is gradually acidified during plasma treatment, usually by 1–3 pH units within 30–60 min, accompanied by the generation of HNO_2_ and HNO_3_ and the dissolution of NO_*x*_, leading to an increase in conductivity, which further reduces the pH of the treated water. This means that initial pH is an incomplete indicator of the actual reaction environment.^248^

Another significant parameter is conductivity, which can also be important in wastewater because high conductivity reduces the production of radicals, such as the production of more chlorine radicals from matrices containing chloride ions, such as Cl˙OH^−^ and Cl_2_^−^. These chlorine radicals have less oxidizing capacity than ˙OH and can affect the treatment process.^249^ The effects of conductivity are further complicated by ionic composition: wastewater whose conductivity is dominated by NaCl behaves differently from wastewater dominated by NaHCO_3_, because the former generates less reactive chlorine radicals, while the latter scavenges ˙OH through carbonate and bicarbonate ions.

The inconsistency of pH and conductivity reporting in the literature for DBD antibiotic treatment is a fundamental limitation: most studies report only the initial pH, fewer than 15% report both initial and final pH and fewer than 5% report conductivity, which makes these parameters among the most poorly reported parameters in the field. DBD systems should therefore report both initial and final pH, conductivity and chloride concentration together, since otherwise comparisons across studies are confounded by shifts in the actual oxidizing environment rather than differences in reactor performance alone.

### 6.5 Initial antibiotic concentration

Initial antibiotic concentration is one of the most consequential and potentially misleading methodological variable in DBD degradation literature because initial concentrations of 10 to 200 mg L^−1^ are employed, which are 3 to 6 orders of magnitude higher than those found in real environmental matrices, where surface water and WWTP effluents contain antibiotics in the ng L^−1^ to low µg L^−1^ range.^5^ Even hospital wastewater, the most concentrated environmental source of antibiotics, only occasionally reaches low mg L^−1^ levels, and then only for individual compounds such as ciprofloxacin.^250^ At the elevated concentrations used in laboratory studies, the antibiotic becomes the dominant ˙OH consumer, which results in artificially high apparent degradation rates because the target compound (antibiotic) competes with other matrix constituents for available reactive species. In real wastewater, natural organic matter, dissolved organics and other co-occurring pharmaceuticals scavenge plasma-generated radicals, leaving a comparatively smaller fraction available for antibiotic oxidation. Spiking trace pharmaceuticals (tens to hundreds of ng L^−1^) into tertiary-treated wastewater has been shown to reduced degradation in a pilot-scale pulsed corona discharge reactor compared to deionized water controls.^251^ Cold plasma treatment of real hospital wastewater showed much lower removal efficiencies than would be predicted from clean water experiments, with matrix organics consuming reactive species that would otherwise target antibiotics.

The practical consequence of such concentration-dependent bias is that energy performance derived from high-concentration lab experiments cannot be meaningfully applied to field conditions. Energy yields (G_50_) spanning 4 orders of magnitude (0.17–7213.23 g kWh^−1^) are observed, which is a variance driven primarily by initial concentration, pollutant recalcitrance, and reactor geometry.^122^ An energy input of 0.26–1.49 kJ mg^−1^ is required for 60% degradation of nine veterinary antibiotics at a concentration of 5 mg L^−1^ in synthetic wastewater, however, these values cannot be extrapolated to performance at µg L^−1^ concentrations,^252^ where dissolved organic carbon consumes most of the generated radicals. In real hospital wastewater, energy requirements reach 0.5–1.0 kWh m^3^ for pharmaceutical removal by pulsed corona discharge.^253^ The field urgently requires a methodological shift toward experiments conducted at environmentally relevant concentrations (ng L^−1^ to µg L^−1^) using sensitive LC-MS/MS detection rather than UV-vis or HPLC-UV analysis in representative water samples.

## 7. Current limitations and practical barriers

Despite the promising lab-scale results, several critical limitations must be addressed for DBD cold-plasma technology to achieve practical implementation. (1) Energy cost and efficiency: energy consumption for antibiotic degradation ranges from 0.3 to 10 kWh m^−3^ depending on reactor design, target concentration and water matrix. (2) Scale-up challenges: DBD plasma from laboratory batch reactors (typically 100–500 mL) to continuous-flow pilot systems (>50 L) introduces several engineering challenges that are not adequately addressed in the current literature (Table 6). First, maintaining a uniform micro-discharge distribution across larger electrode areas requires careful impedance matching and power supply design. Second, gas–liquid mass transfer efficiency decreases as treatment volume increases, unless reactor geometry is specifically optimized (*e.g.*, using falling-film, multi-stage, or bubble configurations). Third, electrode erosion and dielectric degradation become significant at industrial duty cycles, affecting long-term operational costs. The few pilot-scale studies available report energy consumption of 0.5–1.0 kWh m^−3^ for pharmaceutical-grade wastewater, which is competitive with ozonation (0.5–2.0 kWh m^−3^) but still requires validation at full municipal scale. (3) By-product formation: DBD plasma generates transformation products whose toxicity may equal or exceed that of the parent compound. Aromatic amine intermediates from sulfonamide degradation and halogenated by-products from plasma treatment of chloride-containing waters are of particular concern. Fewer than 30% of published studies include ecotoxicity evaluation of treated effluents. (4) Techno-economic and life-cycle assessment (LCA) studies specific to DBD antibiotic treatment are absent in the literature. (5) Lack of standard reporting metrics: the field lacks consensus on how to report energy efficiency (G_50_, EE/O, and kWh m^−3^), treatment dose and water–quality parameters, making cross-study comparison nearly impossible. (6) Real wastewater matrix effects: most studies use spiked deionized water at mg L^−1^ concentrations. Real wastewater contains dissolved organic matter, inorganic ions and competing contaminants that consume 30–70% of generated RONS, substantially reducing treatment efficiency. These factors must be critically considered when interpreting published removal efficiencies and extrapolating laboratory results to real-world applications.

**Table 6:** Comparative energy performance of DBD reactor configurations

Reactor type	Target antibiotic	Energy input (kWh m^−3^)	Removal (%)	EE/O (kWh m^−3^ per order)	Scale	Reference
Coaxial DBD	Tetracycline	0.5–1.5	>90	2.1–4.5	Lab	131
DBD planar falling film	Amoxicillin	0.3–0.8	>95	0.8–1.2	Lab	80
DBD nanosecond-pulsed plasma bubble	Tetracycline	0.4–1.0	97	1.5–3.0	Lab	149
DBD nanosecond-pulsed bubble	Sulfamethoxazole	—	>90	0.29	Lab	176
DBD bubble reactor	Mixed	0.5–1.0	60–80	—	Pilot	142

## 8. Conclusion

Dielectric cold plasma generates multiple RONS *in situ* without chemical additives and achieves high removal efficiencies for the parent compounds of tetracyclines, fluoroquinolones and β-lactams. ARG elimination shows positive results, with reductions in the qPCR-measured abundance of tetR, tetA, blaTEM-1 and similar genes; however, but the presence of impurities and persistent extracellular DNA reduce removal efficiency, and continued spreading of antibiotic resistance through horizontal gene transfer remains unclear. The most significant gap is the continued use of mg L^−1^ initial concentrations in laboratory experiments, which constrains the ability to translate results to real wastewater conditions, where antibiotics are present at ng L^−1^ to low-µg L^−1^ concentrations in complex organic matrices. Additionally, the field still lacks standardized methods for reporting G_50_, EE/O, pH changes and reactor design, making it almost impossible to compare results across studies. Future efforts should shift towards pilot-scale and continuous-flow systems, complemented by techno-economic and life-cycle assessments for hospital and pharmaceutical wastewater, alongside AI-based real-time optimization and renewable-energy-powered solutions to make plasma technology more accessible to communities in greatest need. Taken together, DBD plasma is best viewed not as a single oxidation step but as a modular, versatile and scalable remediation technology with high translational potential for antibiotic wastewater treatment.

## Author contributions

Conceptualization, M. I. A.; methodology, R. A. and A. S.; software, R. A., A. S. and J. S.; validation, M. S. and A. J.; formal analysis, H. W., A. J. and A. S.; investigation, M. I. A., A. J. and J. S.; resources, M. I. A., A. J. and M. S.; data curation, H. W. and J. S.; writing – original draft preparation, R. A. and A. S.; writing – review and editing, R. A.; visualization, R. A.; supervision, M. I. A., A. J., M. S. and H. W; project administration, M. I. A., A. J., M. S. and H. W; and funding acquisition, H. W.

## Conflicts of interest

The authors declare no potential conflicts of interest.

## Data Availability

No primary research results, software or code have been included, and no new data were generated or analyzed as part of this review.
